# Complex evolutionary history of the Mexican stoneroller *Campostoma ornatum *Girard, 1856 (Actinopterygii: Cyprinidae)

**DOI:** 10.1186/1471-2148-11-153

**Published:** 2011-06-04

**Authors:** Omar Domínguez-Domínguez, Marta Vila, Rodolfo Pérez-Rodríguez, Nuria Remón, Ignacio Doadrio

**Affiliations:** 1 Laboratorio de Biología Acuática, Facultad de Biología, Universidad Michoacana de San Nicolás de Hidalgo, Morelia, Michoacán, México; 2 Department of Molecular and Cell Biology, Faculty of Sciences, University of A Coruña, Campus Zapateira, E-15008 A Coruña, Spain; 3 Departamento de Zoología, Instituto de Biología, Universidad Nacional Autónoma de México, C.P. 04510, Apartado Postal 70-153, México, D.F., México; 4 Departamento de Biodiversidad y Biología Evolutiva, Museo Nacional de Ciencias Naturales, CSIC, c/José Gutiérrez Abascal 2, E-28006 Madrid, Spain

## Abstract

**Background:**

Studies of the phylogeography of Mexican species are steadily revealing genetic patterns shared by different species, which will help to unravel the complex biogeographic history of the region. *Campostoma ornatum *is a freshwater fish endemic to montane and semiarid regions in northwest Mexico and southern Arizona. Its wide range of distribution and the previously observed morphological differentiation between populations in different watersheds make this species a useful model to investigate the biogeographic role of the Sierra Madre Occidental and to disentangle the actions of Pliocene tecto-volcanic processes *vs *Quaternary climatic change. Our phylogeographic study was based on DNA sequences from one mitochondrial gene (*cytb*, 1110 bp, n = 285) and two nuclear gene regions (S7 and RAG1, 1822 bp in total, n = 56 and 43, respectively) obtained from 18 to 29 localities, in addition to a morphological survey covering the entire distribution area. Such a dataset allowed us to assess whether any of the populations/lineages sampled deserve to be categorised as an evolutionarily significant unit.

**Results:**

We found two morphologically and genetically well-differentiated groups within *C. ornatum*. One is located in the northern river drainages (Yaqui, Mayo, Fuerte, Sonora, Casas Grandes, Santa Clara and Conchos) and another one is found in the southern drainages (Nazas, Aguanaval and Piaxtla). The split between these two lineages took place about 3.9 Mya (CI = 2.1-5.9). Within the northern lineage, there was strong and significant inter-basin genetic differentiation and also several secondary dispersal episodes whit gene homogenization between drainages. Interestingly, three divergent mitochondrial lineages were found in sympatry in two northern localities from the Yaqui river basin.

**Conclusions:**

Our results indicate that there was isolation between the northern and southern phylogroups since the Pliocene, which was related to the formation of the ancient Nazas River paleosystem, where the southern group originated. Within groups, a complex reticulate biogeographic history for *C. ornatum *populations emerges, following the taxon pulse theory and mainly related with Pliocene tecto-volcanic processes. In the northern group, several events of vicariance promoted by river or drainage isolation episodes were found, but within both groups, the phylogeographic patterns suggest the occurrence of several events of river capture and fauna interchange. The Yaqui River supports the most diverse populations of *C. ornatum*, with several events of dispersal and isolation within the basin. Based on our genetic results, we defined three ESUs within *C. ornatum *as a first attempt to promote the conservation of the evolutionary processes determining the genetic diversity of this species. They will likely be revealed as a valuable tool for freshwater conservation policies in northwest Mexico, where many environmental problems concerning the use of water have rapidly arisen in recent decades.

## Background

The relative role of geology and ecology in driving lineage differentiation, and ultimately speciation, has been an important topic in evolutionary biology since the 19^th ^century. At present, it is a thriving field of research aiming to disentangle the relative importance of glacial refugia, extrinsic environmental factors and species interactions, aided to the ability of locating species boundaries, phylogeographical breaks and hybrid zones [1].

Montane ecosystems are areas of high endemism, and the mechanisms driving this endemicity have been receiving increasing attention. For instance, endemism in tectonically active regions should reflect cladogenesis within the montane region, rather than the contraction of geographic range from a much larger region ([2], but see also [3]). Additionally, climatic factors have been rejected as the only explanation for the species richness of taxa within small to medium-sized geographic ranges; thus, both geological and evolutionary processes must be considered [4]. Molecular studies of montane Mexican taxa often revealed complex phylogeographical patterns, and those high levels of genetic divergence suggest an underestimation of the level of endemism in the Mexican highlands, implying that more surveys in other co-distributed taxa are needed to achieve a better understanding of the evolutionary drivers of diversification in these regions [5].

What is clear at the present time is that the evolutionary history of many Meso- and North American taxa is linked to the severe geological (tectonic and volcanic) and environmental changes that occurred during the Neogene and Quaternary periods [3,6,7]. The challenge now is to distinguish the relative contribution of each factor to the observed phylogeographic pattern. The number of studies that have been conducted in the Mexican endemic-rich area of the northern sierras is lower than the number of studies of the Trans-Mexican Volcanic Belt (TMVB) (e.g., [8]). Therefore, one of the main contributions of the present work is to investigate the biogeographic role of the Sierra Madre Occidental (SMOC) using an endemic freshwater species. The SMOC may have acted as either a barrier where current admixture of lineages can occur, as a corridor for range expansion or as a centre of diversification, promoting allopatric differentiation.

### Biogeographic role of the Sierra Madre Occidental

The SMOC is a large volcanic plateau in western Mexico that extends parallel to the Pacific coastline for more that 1200 km from the US-Mexico border (31° N) to the TMVB (21° N). The topography of the northern Sierra Madre is characterised by a high average elevation (1909 m). In contrast to other North American mountain systems, the topographic relief of the SMOC is not the product of elevated mountain ranges, but rather of incised canyons. The western edge is quite steep, while the eastern topographic gradients from the Sierra Madre into the central Mexican Plateau are relatively smooth [9].

The SMOC is thought to form an important corridor of dispersal for tropical flora and fauna moving in response to climatic change [10,11]. This mountain system is one of the 14 biogeographic provinces of Mexico and one of the five biogeographic provinces of the so-called Mexican Transition Zone, mainly defined by the plant and animal taxa that are found above 1000 m. This province has the highest Nearctic influence [11].

The SMOC is the result of Cretaceous-Cenozoic magmatic and tectonic episodes related to the subduction of the Farallon plate beneath North America and to the opening of the Gulf of California. During the Oligocene, extensional tectonics (processes associated with the stretching of the lithosphere) began in the entire eastern half of the SMOC, migrating towards the west during the Miocene [12]. The resulting post-Oligocene river capture has been inferred from geological data from the southern flank of the Tepic-Zacoalco rift (southern SMOC), although more recent piracy phenomena have been reported in other areas ([13] and references therein) or suggested by phylogeographic studies (e.g., [14]).

To the best of our knowledge, the phylogeography of ichthyofauna endemic to the SMOC has not been thoroughly investigated, although population genetics surveys have been conducted for *Oncorhynchus chrysogaster *([15] and references therein) and *Poeciliopsis sonorensis *[16]. As pointed out by recent literature [5], there are only a limited number of surveys on the genetic structure of endemic species from the North and Mesoamerican mountains (e.g., [17,18]).

We consider two alternative biogeographic scenarios. Given the fact that the SMOC acts as a biogeographic barrier for many taxa, a plausible first hypothesis would be that the phylogeographic pattern of *Campostoma ornatum *would result from the current admixture of formerly West and East lowland lineages isolated during the Pleistocene interglacial stages [19-22]. If so, we would expect to find major lineages located on the northwestern and southeastern slopes and an altitudinal gradient of haplotype diversity. A second putative role for the SMOC would be as a centre of diversification, related to a working hypothesis of a highland distribution of *C. ornatum *predating the Quaternary ice ages. Under such a scenario, deep genetic and morphological divergences between the study species would have been shaped by geological processes rather than by climatic oscillations. Additionally, extensional tectonics causing river piracy (a geomorphological phenomenon occurring when a stream or river drainage system is diverted from its own bed and connects with the one of a neighbouring stream) would cause a mismatch between the actual conformation of river drainages and genetic patterns.

### Study species

**Figure 1:**
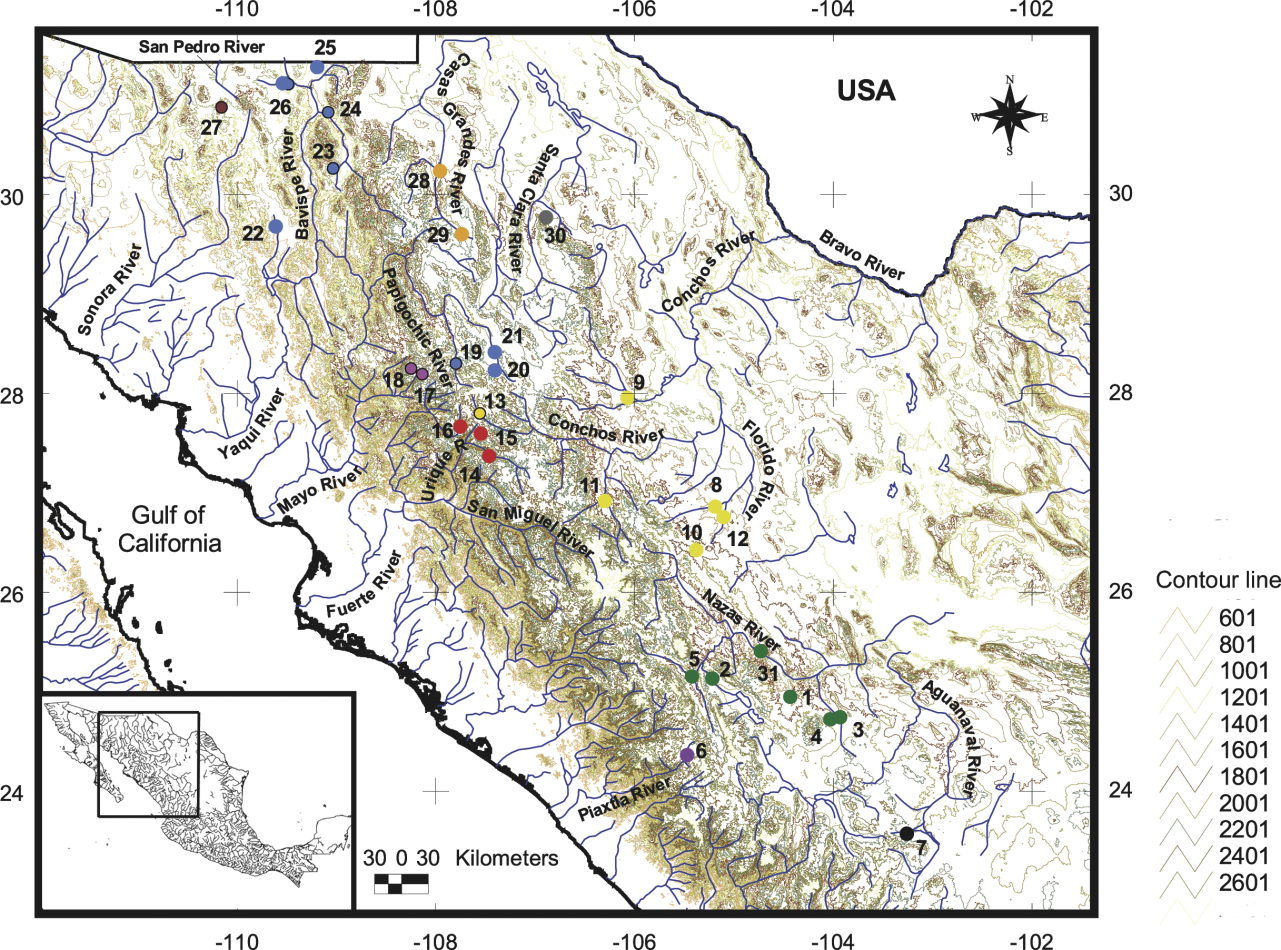
**Sampling sites in northwestern Mexico from which the *Campostoma ornatum *specimens were obtained**. Numbers on the map correspond to the numbers in Table 1. 1: El Cuarto, 2: Olote, 3: Peñon Blanco, 4: Covadonga, 5: Atotonilco, 6: La Quinta, 7: Sain Alto, 8: Rio Primero, 9: Satevo, 10: Ocampo, 11: Porvenir, 12: Coronado, 13: Bocoyna, 14: Urique, 15: Rimichurachi, 16: Oteros, 17: Basaseachic, 18: Concheño, 19: La Tauna, 20: Tomochic, 21: Papigochic, 22: Terapa, 23: Huachinera, 24: Hondables, 25: Agua Prieta, 26: Cabullona, 27: Ojo de Agua, 28: Casas Grandes, 29: Ignacio Zaragoza and 30: Santa Clara. Colours of circles are related to group colours in Figure 2. This map has contours marked at 200 m intervals.

To study the biogeographic role of the SMOC on its ichthyofauna, we selected the Mexican stoneroller, *Campostoma ornatum*, as our model organism. The Mexican stoneroller is endemic to the central and northern sectors of the SMOC and its northern fringes (Madrean Sky-Islands). It is found on both slopes of the SMOC: in the Bravo and Conchos Rivers, as well as in the Casas Grandes and Santa Clara (also known as El Carmen) interior drainages and the upper parts of the Yaqui, Mayo and Fuerte north-western Pacific River basins. The southernmost basins where *C. ornatum *is present are the interior Nazas and Aguanaval Rivers and the Pacific Piaxtla River basin [23] (Figure 1).

The widespread distribution of *C. ornatum *is thought have been shaped by tectonics (stream capture between adjacent drainages) and climatic changes (dispersion via periodically formed floodplains or the connection or succession of pluvial lakes during glacial stages) [24]. Its distribution area (the elevation range of the species is 800 - 2000 m a.s.l.) coincides with a biodiversity hotspot, the the Madrean Pine-Oak Woodlands [25].

Several cyprinid species for which deep intraspecific genetic divergence has been previously reported exist in large areas severely influenced by both tecto-volcanic activity and climatic changes [26-28]. In fact, some of those widespread species have been split into more than one taxon, in light of their genetic divergence and morphological differentiation [14,29,30]. This was the case for *Campostoma anomalum*, which was previously regarded as a complex of three widespread and morphologically divergent subspecies, whose five distinct evolutionary lineages warranted species status [31,32]. The species *Campostoma ornatum*, also with a wide distribution range and morphological variation, is also likely to show deep intraspecific genetic differentiation because populations from southern drainages (Nazas, Aguanaval and Piaxtla rivers) show a differentiated morphotype [24] and there is remarkable endemicity in the ichthyofauna of the Nazas river [33,34].

### Conservation perspective

Phylogeography also has an applied perspective from the conservation point of view. The genetic and morphological information obtained in the present work will be examined to define, if appropriate, evolutionarily significant units (ESUs) within *Campostoma ornatum*. The need to preserve the loss of *Campostoma *lineages restricted to areas where aquatic biodiversity is imperilled was already highlighted [31]. In addition, the populations of the Mexican stoneroller in the USA declined during the 20^th ^century [35] and there were plans to perform reintroductions from Mexico [36].

At the present time, an ESU is essentially acknowledged to be a population or a group of populations that merit the separate management or priority of conservation because it is highly distinctive, according to the following criteria: (i) reproductive isolation and adaptation, (ii) reciprocal monophyly and (iii) "exchangeability" of populations. Concordance across multiple data types (e.g., genetic, morphological and geographic) is, of course, desirable (revised by [37]). Defining conservation units in endemic species may be of particular interest in densely populated countries, such as Mexico. This is because environmental problems concerning the use of water rapidly arise in semiarid regions, such as northwest Mexico [38,39], where the demand for water resources has dramatically increased in recent decades [40,41].

In the present study we aim to infer the evolutionary history of *C. ornatum *throughout its distribution area in order to determine the role of the SMOC on the evolutionary history of *C. ornatum *and to disentangle the action of tecto-volcanic processes vs. climatic change. To do this, we applied phylogenetic and population genetic methods to sequence data obtained for a mitochondrial gene (cytochrome b, *cytb*) and two nuclear fragments (RAG1 and S7) from 18 to 29 locations within the distribution area of *C. ornatum*. In addition, we included several meristic characters to first corroborate the morphological variation pattern previously observed [24] and second to investigate the putative association between genetic and phenotypic data. In line with our main objective, we also addressed the following question from the conservation perspective: do any of the populations/lineages sampled in different drainages deserve to be categorised as an (ESU) under the Adaptive Evolutionary Conservation (AEC) framework [42]?

## Results

### Genetic diversity

Sixty haplotypes were obtained by sequencing the *cytb *gene in the 285 samples collected in the current study (Table 1 and Figure 1). These haplotypes were defined by 160 variable sites within a 1110-bp sequence fragment (total number of mutations = 169). Twenty-three of those sites were singletons, and 137 substitutions were parsimony informative. Twenty-two changes involved amino acid replacements, and 147 were synonymous. Overall, haplotype diversity (*h*) was 0.964 ± 0.003 (standard deviation), nucleotide diversity (*π*) = 0.032 ± 0.001, the average number of nucleotide differences (*k*) = 35.041 and Tajima's *D *= +0.895 (not significant) (Tables 2 and 3).

**Table 1:** Sampled localities and material analysed.

Population	Locality and elevation (metres above sea level)	Drainage	Voucher	Genbank accession number		
				
				cyt *b*	S7	RAG1
1.Cuarto (CUA)	San Juan River in the Cuarto area, San Juan del Río town, Durango, Mex. (1525)	Nazas	CPUM1530, CPUM6701-CPUM6709	JF343058 (8), JF343059 (2)	JF343112 (1)	
2.Olote (OLO)	Ramos River, El Olote town, Durango, Mex. (1652)	Nazas	CPUM6838-CPUM6843, CPUM6845-CPUM6849	JF343039 (2), JF343041 (4), JF343042 (1), JF343070 (1), JF343071 (1), JF343072 (1), JF343073 (1)	JF343111 (1)	JF343114 (2
3.Peñón Blanco (PBY)	Stream outskirts of Peñón Blanco Town, Durango, Mex. (1739)	Nazas	CPUM1654, CPUM6554-CPUM6563	JF343057 (10), JF343078 (1)		
4.Covadonga (COV)	Covadonga River, at Peñon Blanco Town, Durango, Mex. (1730)		CPUM6889-CPUM6890,			
		Nazas	CPUM6896-CPUM6898	JF343057 (5)	JF343109 (1)	JF343114 (1)
5.Atotonilco (ATO)	Ramos River, Atotonilco town, Durango, Mex. (1693)	Nazas	CPUM6727-CPUM6730, CPUM6752-CPUM6757	JF343039 (1), JF343040 (1), JF343041 (7), JF343042 (1)	JF343113 (3)	JF343114 (2)
						
6.Quinta (QNT)	Piaxtla River, La Quinta town, Durango, Mex. (2391)	Piaxtla	CPUM8902-CPUM8910, CPUM8927	JF343042 (10)	JF343110 (1), JF343113 (2)	
7.Sain Alto (SAA)	Sain Alto River near Atotonilco, Zacatecas, Mex. (1999)	Aguanaval	UAIC 7895.01	DQ324062		
8.Río Primero (RIP)	Primero River, road Torreón de Mata town, Chihuahua, Mex. (1720)	Conchos	CPUM1917-CPUM1919	JF343055 (3)	JF343105 (1), JF343106 (1)	JF343116 (1), JF343120 (1)
9.Satevo (SAT)	River at San Francisco Javier de Satevo town, Chihuahua, Mex. (1450)	Conchos	CPUM1993-CPUM1995	JF343084 (2), JF343085 (1)		
10.Ocampo (OCA)	Villa Ocampo, Chihuahua, Mex. (1832)	Conchos	CPUM1955-CPUM1957	JF343055 (2), JF343066 (1)	JF343104 (1)	JF343116 (1)
11.Porvenir (POR)	El Porvenir River, road La Magdalena town-Balleza town, Chihuahua, Mex. (1579)	Conchos	CPUM6995-CPUM7004	JF343055 (1), JF343056 (1), JF343079 (8)	JF343104 (1)	JF343116 (1)
12.Coronado (COR)	Florido River at Villa Coronado town, Chihuahua, Mex. (1511)	Conchos	CPUM6911-CPUM6921	JF343055 (10), JF343056 (1)	JF343107 (1)	JF343116 (1)
13.Bocoyna (BCY)	Conchos River at Bocoyna town, Mex. Chihuahua, Mex. (2219)	Conchos	CPUM7231-CPUM7240	JF343045 (10)	JF343106 (1)	
14.Urique (URI)	Urique River road Guachochi town-Creel town, Chihuahua, Mex. (1647)	Fuerte	CPUM7077-CPUM7088	JF343045 (12)		
15.Rimichurachi (RIM)	Cuzarare stream, at Rimichurachi town, S of Creel, Chihuahua, Mex. (2196)	Fuerte	CPUM7135-CPUM7136, CPUM7138-CPUM7145	JF343081 (2), JF343082 (5), JF343083 (3)		
16.Oteros (OTE)	Oteros River Southwest to Creel town, Chihuahua, Mex. (2065)	Fuerte	CPUM7169, CPUM7172, CPUM7175-CPUM7182, CPUM7229-CPUM7230	JF343074 (11), JF343075 (1)	JF343106 (1)	JF343116 (1)
17.Basaseachic (BAS)	Aguacaliente River at Basaseachic town, Chihuahua, Mex. (2005)	Mayo	CPUM7392-CPUM7401	JF343043 (9), JF343044 (1)	JF343103 (1)	JF343116 (1)
18.Concheño (CON)	Concheño River, road El Placer town-Ocampo town, Chihuaua, Mex. (2225)	Mayo	CPUM7432-CPUM7436, CPUM7438-CPUM7443	JF343043 (11)		
19.Tauna (TAU)	River at La Tauna town, Chihuahua, Mex. (1764)	Yaqui	CPUM7462-CPUM7471	JF343043 (4), JF343086(5), JF343087 (1)		
20.Tomochic (TOM)	Tomochic River at Tomochic town, Chihuahua, Mex. (2210)	Yaqui	CPUM7543-CPUM7550	JF343076 (4), JF343092 (1), JF343093 (1), JF343094 (1), JF343095 (1)		
21.Papigochic (PAP)	Río Papigochic at ranch near to Pahuirachic, Chihuahua, Mex. (1764)	Yaqui	CPUM7328-CPUM7339	JF343076 (11), JF343077 (1)	JF343101 (1)	JF343119 (1)
22.Terapa (TER)	South of Moctezuma, Sonora, Mex. (558)	Yaqui	CPUM7883-CPUM7889, CPUM7891-CPUM7894	JF343088 (3), JF343089 (6), JF343090 (1), JF343091 (1)		
23.Huachinera (HUA)	Bavispe River, Huachinera town, Sonora, Mex. (1062)	Yaqui	CPUM7669-CPUM7670, CPUM7674-CPUM7676, CPUM7678-CPUM7682	JF343064 (10)		
24.Hondables (HON)	Bavispe River at Los Hondables, Morelos, Sonora, Mex. (857)	Yaqui	CPUM7800-CPUM7809	JF343064 (9), JF343065 (1)	JF343100 (2)	JF343117 (1)
25.Agua Prieta (PRI)	Stream road Agua Prieta city-Janos city, Sonora, Mex. (1113)	Yaqui	CPUM7768-CPUM7784	JF343047 (4), JF343048 (8), JF343080 (5)	JF343100 (13)	JF343117 (7), JF343118 (4)
26.Cabullona (CAB)	Cabullona River, road Agua Prieta city-Nacozari de García city, Sonora, Mex. (1134)	Yaqui	CPUM7843-CPUM7857	JF343046 (5), JF343047 (3), JF343048 (6), JF343049 (1)	JF343099 (1), JF343100 (13)	JF343117 (10), JF343118 (1)
27.Ojo de Agua (OJO)	Spring at road Cananea city-Bacoachi town, Sonora, Mex. (1386)	Sonora	CPUM7827-CPUM7836	JF343067 (3), JF343068 (2), JF343069 (5)	JF343108 (3)	JF343118 (3),
28.Casas Grandes (GRA)	Casas Grandes River at Hacienda San Diego, S of Casas Grandes city, Chihuahua, Mex. (1510)	Casas Grandes	CPUM7752-CPUM7761	JF343060 (1), JF343061 (6), JF343062 (1), JF343063 (2)	JF343100 (4)	JF343117 (2)
29.I. Zaragoza (ZAR)	Casas Grandes River at Ignacio Zaragoza town, Chihuahua, Mex. (2077)	C. Grandes	CPUM7562-CPUM7571	JF343060 (4), JF343096 (1), JF343097 (4), JF343098 (1)		
30.Santa Clara (CLA)	Santa Clara River at San Lorenzo, East of Buenaventura, Chihuahua, Mex. (1524)	Santa Clara	CPUM7643-CPUM7644	JF343050 (4), JF343051 (1), JF343052 (1), JF343053 (4), JF343054 (1)	JF343102 (2)	JF343115 (2)

Acronyms used for each location as well as number of individuals from the same site sharing haplotype are indicated in parentheses.

**Table 2:** Measurements of genetic diversity for *Campostoma ornatum *as revealed by 1110 bp of the mitochondrial *cytb *and calculated according to sampling location.

Locality	River basin	*n*	*NH*	*S*	*h*	*R*	*π*	*k*	Tajima's *D*
Atotonilco (ATO)	Nazas	10	4	10	0.533 ± 0.180	0.892	0.0020 ± 0.0012	2.267	-1.590^a^
Basaseachic (BAS)	Mayo	10	*2*	*1*	0.2 ± 0.154	0.300	0.0002 ± 0.0001	0.2	-1.112
Bocoyna (BCY)	Conchos	10	*1*	*0*	*0*	*0*	*0*	*0*	n.a.
Cabullona (CAB)	Yaqui	**15**	4	**44**	0.733 ± 0.067	1.268	**0.0174 ± 0.0021**	**19.333**	**+1.837^a^**
Santa Clara (CLA)	Santa Clara	10	**5**	7	**0.8 ± 0.1**	**1.442**	0.0019 ± 0.0004	2.089	-0.659
Concheño (CON)	Mayo	11	*1*	*0*	*0*	*0*	*0*	*0*	n.a.
Villa Coronado (COR)	Conchos	11	*2*	4	0.182 ± 0.144	0.273	0.0007 ± 0.0005	0.727	*-1.712^a^*
Covadonga (COV)	Nazas	*5*	*1*	*0*	*0*	*0*	*0*	*0*	n.a.
El Cuarto (CUA)	Nazas	10	*2*	*1*	0.356 ± 0.159	0.533	0.0003 ± 0.0001	0.356	+0.015
Casas Grandes (GRA)	Casas Grandes	10	4	4	0.644 ± 0.152	1.100	0.0012 ± 0.0003	1.289	-0.339
Los Hondables (HON)	Yaqui	10	*2*	*1*	0.2 ± 0.154	0.300	*0.0002 ± 0.0001*	0.2	-1.112
Huachinera (HUA)	Yaqui	10	*1*	*0*	*0*	*0*	*0*	*0*	n.a.
Villa Ocampo (OCA)	Conchos	*3*	*2*	5	0.667 ± 0.314	1.000	0.003 ± 0.0014	3.333	n.a^b^.
Ojo de Agua (OJO)	Sonora	10	3	3	0.689 ± 0.104	1.158	0.0011 ± 0.0003	1.267	+0.699
El Olote (OLO)	Nazas	11	**7**	16	**0.873 ± 0.089**	**1.642**	0.0044 ± 0.0011	4.836	-0.511
Oteros (OTE)	Fuerte	12	*2*	*1*	0.167 ± 0.134	0.250	0.0001 ± 0.0001	0.167	-1.140
Papigochic (PAP)	Yaqui	12	*2*	2	*0.167 ± 0.134*	*0.250*	0.0003 ± 0.0002	0.333	-1.4514
Peñón Blanco-Yerbaniz (PBY)	Nazas	11	*2*	*1*	*0.182 ± 0.144*	*0.273*	0.0002 ± 0.0001	0.182	-1.128
Porvenir (POR)	Conchos	10	3	4	0.378 ± 0.181	0.600	0.0007 ± 0.0004	0.8	-1.667^a^
Agua Prieta (PRI)	Yaqui	**17**	3	**42**	0.676 ± 0.064	1.132	**0.0166 ± 0.0023**	**18.382**	**+1.847^a^**
La Quinta (QNT)	Piaxtla	10	*1*	*0*	*0*	*0*	*0*	*0*	n.a.
Rimichurachi (RIM)	Fuerte	10	3	2	0.689 ± 0.104	1.158	0.0009 ± 0.0001	1.022	+1.439
Primero (RIP)	Conchos	*3*	*1*	*0*	*0*	*0*	*0*	*0*	n.a^b^.
Satevó (SAT)	Conchos	*3*	*2*	*1*	0.667 ± 0.314	1.000	0.0006 ± 0.0003	0.667	n.a^b^.
La Tauna (TAU)	Yaqui	10	3	3	0.644 ± 0.101	1.050	0.0008 ± 0.0003	0.933	-0.431
Terapa (TER)	Yaqui	11	4	7	0.673 ± 0.123	1.145	0.0018 ± 0.0007	1.964	-0.7238
Tomochic (TOM)	Yaqui	8	5	4	0.786 ± 0.151	1.429	0.0009 ± 0.0002	1	-1.5347^a^
Urique (URI)	Fuerte	12	1	0	0	0	0	0	n.a.
Ignacio Zaragoza (ZAR)	Casas Grandes	10	4	6	0.733 ± 0.101	1.267	0.0017 ± 0.0004	1.867	-0.4959

Standard deviations after symbol (±): *n*, number of individuals; *NH*, number of haplotypes; *S*, number of segregating sites; *h*, haplotype diversity; *R*, allelic richness after rarefaction; *π*, nucleotide diversity per site; and *k*, average number of nucleotide differences. The two largest values for each variable are highlighted in bold, whereas the two minimum ones are marked in italics.

a 0.1 >*p *> 0.05

b minimum number of individuals required to perform the test = 4

**Table 3:** Genetic diversity measurements for the eight *cytb *phylogroups obtained by the 95% SP unconnected subnetworks for *Campostoma ornatum*.

Phylogroup	*n*	*S*	*H*	*Hd*	*π*	*k*	*F*_S _(95% C.I.)	*R2 *(95% C.I.)
I	44	5	6	0.683	**0.001**	*0.15*	-0.77 (-3.66, 3.88)	0.11 (0.06, 0.23)
II	10	*3*	*3*	0.689	**0.001**	1.27	0.95 (-1.96, 3.34)	0.21 (0.14, 0.3)
III	30	7	6	*0.655*	*0.002*	0.8	0.08 (-3.73, 4.28)	0.14 (0.07, 0.2)
IV	10	7	5	0.8	**0.1**	2.09	-0.50 (-3.2, 3.59)	0.14 (0.11, 0.26)
V	10	*1*	*2*	*0.56*	*0.0005*	*0.56*	1.09 (-0.34, 1.09)	0.28 (0.18, 0.3)
VI	76	**39**	**15**	**0.825**	0.009	**9.94**	4.16 (-9.36, 7.18)	0.12 (0.05, 0.16)
VII	48	**14**	**11**	**0.801**	*0.002*	**2.16**	-2.51 (-5.99, 5.48)	0.07 (0.05, 0.18)
VIII	57	20	12	0.838	0.005	5.75	1.55 (-6.76, 5.68)	0.14 (0.05, 0.17)

*n*, number of individuals; *NH*, number of haplotypes; *S*, number of segregating sites; *h*, haplotype diversity; *R*, allelic richness after rarefaction; *π*, nucleotide diversity per site; and *k*, average number of nucleotide differences, the two largest values for each variable are highlighted in bold, whereas the two minimum ones are marked in italics.

Results for Fu's (*F*_S_) and Ramos-Onsins & Rozas' (*R2*) tests for detecting population growth are also shown. Values close to significance are displayed underlined.

We sequenced the S7 nuclear gene in 56 specimens from 19 locations, representing all surveyed river basins. Twenty-eight of these individuals had between one and seven heterozygous positions. After haplotype reconstruction, 24 variants were defined by 36 variable sites (*plus *four indels) along the 843 bp of the final alignment (37 mutations in total). Two of the variable sites were singletons, and 34 substitutions were parsimony informative. Overall, haplotype diversity (*h*) was 0.862 ± 0.02, nucleotide diversity (*π*) = 0.008 ± 0.001, the average number of nucleotide differences (*k*) = 6.99 and Tajima's *D *= -0.066 (not significant). Fourteen sites showed insertion/deletion variation. Indel polymorphism was estimated in four indel events and haplotypes (average indel length event = 3.5, indel haplotype diversity = 0.169). When heterozygous positions are coded as unknown but gaps are kept as the fifth state, the number of haplotypes decreases to 15, defined by 18 variable sites (plus indels). Haplotype diversity (*h*) was reduced to 0.632 ± 0.068, nucleotide diversity (*π*) = 0.0047 ± 0.0008, the average number of nucleotide differences (*k*) = 3.84 and Tajima's *D *= -0.0602 (not significant). Three of the variable sites correspond to singletons, and 15 substitutions were parsimony informative.

The populations from Santa Clara (Santa Clara River basin) and El Olote (Nazas River basin) were the most diverse, as measured by the number of haplotypes, gene diversity and allelic richness (Table 2). Seven populations, scattered along the Fuerte, Mayo, Nazas, Yaqui and Conchos basins, contained only one haplotype (Table 2). Divergent haplotypes were found in Cabullona (CAB) and Agua Prieta (PRI), both in the Yaqui River basin, as shown by the number of segregating sites (*S*) and average number of nucleotide differences (*k*) (Table 2). The possibility of admixture of lineages led us to increase the number of sequenced individuals at these two sites to 15 and 17, respectively. When genetic diversity parameters were calculated according to the phylogroups indicated by the 95% SP unconnected subnetworks (see below), phylogroups VI (Yaqui, San Bernardino and Mayo River locations) and VIII (Nazas, Aguanaval and Piaxtla River locations) were the most diverse, whereas phylogroup V (Cabullona and San Bernardino River locations) was the least diverse (Table 3).

Twenty-two of the 43 individuals for which the RAG1 nuclear gene was sequenced had between one and three heterozygotic positions. After haplotype reconstruction, 12 variants were defined by 12 variable sites along the 979 bp of the final alignment (total number of mutations = 12). No gaps were found. Overall, haplotype diversity (*h*) was 0.847 ± 0.021, nucleotide diversity (*π*) = 0.00228 ± 0.00014, the average number of nucleotide differences (*k*) = 2.23 and Tajima's *D *= -0.166 (not significant). One variable site was a singleton and 11 substitutions were parsimony informative. Eight substitutions corresponded to synonymous changes, with the other four implicating amino acid replacements. If heterozygous positions were coded as unknown, six haplotypes were obtained for the 44 individuals mentioned above. These haplotypes were defined by six variable sites (total number of mutations = 6). Two of these sites were singletons, and the remaining four substitutions were parsimony informative. Two changes involved amino acid replacement, and four were synonymous. Overall, haplotype diversity (*h*) was 0.568 ± 0.077 (standard deviation), nucleotide diversity (*π*) = 0.00087 ± 0.00018, the average number of nucleotide differences (*k*) = 0.8425 and Tajima's *D *= -1.017 (not significant).

### Phylogenetic reconstruction

**Figure 2:**
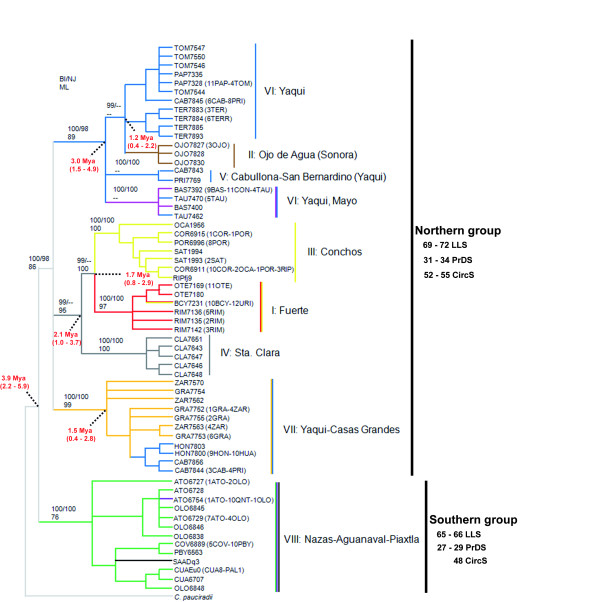
**Phylogenetic reconstruction of *cytb *gene haplotypes**. BI tree for the *Campostoma ornatum *mtDNA haplotypes sampled in this study. Posterior probabilities for the BI (top left), bootstrap support for NJ (top right) and for ML (bottom) are given for the relevant nodes. Numbers in red represent the dates (Mya) and confidence intervals obtained with BEAST. Branch colours mirror codes for sampling sites (Figure 1). Range for mode values of three informative meristic characters (LLS: lateral line scales; PrDS: predorsal scales; CircS: circumferential scales) is displayed on the right.

Phylogenetic hypotheses based on *cytb *(Figure 2) and S7 (not shown) recovered two highly supported main clades. The first group included the Atlantic drainage of Conchos, the interior drainages of Santa Clara, Casas Grandes and the western Pacific drainages Yaqui, Fuerte, Sonora and Mayo. A second clade clustered the southern populations of *C. ornatum*: the interior drainage of Nazas and Aguanaval Rivers as well as the Piaxtla basin (Figure 2). However, the phylogenetic tree based on RAG1 (not shown) did not reveal such a monophyletic group for this "southern" group of populations, even after several other North American cyprinids were used as outgroups.

Within the northern lineage, several incongruities were present among the three loci. The nuclear loci showed a large basal polytomy for all groups, as expected for fewer variable markers. The better resolved mitochondrial topology (Figure 2), largely congruent with all of the analyses conducted (NJ, BI and ML), showed a large polytomy forming three groups: one included populations within the Yaqui and Sonora drainages; a second group comprised populations within the Conchos, Fuerte and Santa Clara drainages; and a third group was formed by populations within Yaqui and Casas Grandes drainages. This pattern did not recover the geographic pattern of the drainages, as only the Santa Clara River drainage conformed to a monophyletic group. All of the other drainages showed mixed haplotypes (Figure 2), and the southern population showed mixed haplotypes between drainages.

**Figure 3:**
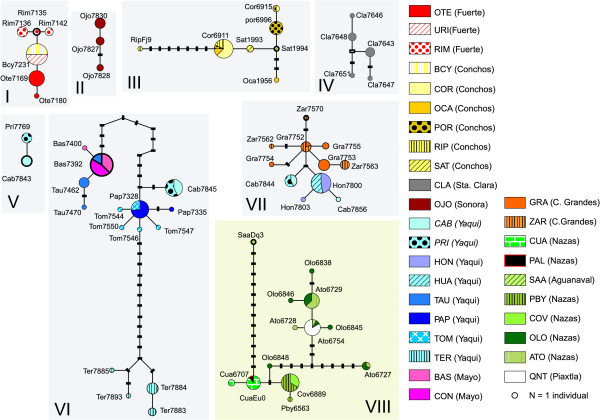
**Statistical parsimony (95%) network of 60 *cytb Campostoma ornatum *haplotypes**. Eight unconnected subnetworks were obtained (95%, connection limit = 14). Abbreviations correspond to the names shown in Table 1. River basins are in parentheses. Circle size reflects the frequency of each haplotype. Solid lines connecting each pair of haplotypes represent a single mutational event, regardless of their length. Small black rectangles represent missing or theoretical haplotypes. Roman numbers identify each subnetwork. Haplotype names are displayed beside each circle. Localities CAB and PRI (italics) contained individuals that clustered in two different subnetworks. Subnetwork VIII, shadowed in light green, was the most differentiated. The basal haplotype of each subnetwork is highlighted with a thicker circumference. Haplotypes RipFj9, SaaDq3 and CuaEu0 were retrieved from Genbank (accession numbers FJ913814, DQ324062 and EU082476, respectively).

Applying the 95% statistical parsimony (SP) criterion to the mitochondrial dataset results in eight unconnected subnetworks (Figure 3). Haplotypes differing by up to 14 mutations could be connected, with 95% confidence that multiple substitutions did not occur at any particular nucleotide position. Overall, subnetwork VIII (Nazas-Aguanaval-Piaxtla River basins) was the most differentiated, as revealed by the fact that it remained unconnected from the other seven subnetworks until a step limit of 50 was enforced. Overall, haplotype CUA6707, from subnetwork VIII, was the most basal, as it has the fewest number of substitutions, when compared to the root (*Campostoma oligolepis*).

Most of the subnetworks were composed of haplotypes found in more than one drainage; only Santa Clara (CLA) (subnetwork IV) and Ojo de Agua (OJO) (within the Sonora basin; subnetwork II) contained private haplotypes not shared with any other subnetwork or basin (Figure 3). Although subnetworks III and V were composed of haplotypes found in only one basin (Conchos and Yaqui), haplotypes from Conchos also appeared in subnetwork I, whereas haplotypes from Yaqui were also present in subnetworks VI and VII (Figure 3). We found that haplotypes from the Tomochic and Papigochic sites (subnetwork VI) grouped in a star-like shape, as did haplotypes from the Casas Grandes (GRA) and Ignacio Zaragoza (ZAR) populations (within subnetwork VII).

Several divergent lineages were found in two particular locations: some haplotypes from Cabullona (CAB) and Agua Prieta (PRI) (both in the Yaqui River basin) formed subnetwork V, whereas other haplotypes from CAB and PRI were connected to different river basins (Mayo from subnetwork VI, Casas Grandes from subnetwork VII). This points to a distinct evolutionary history for each of those mtDNA lineages currently sympatric within the Yaqui River basin.

**Figure 4:**
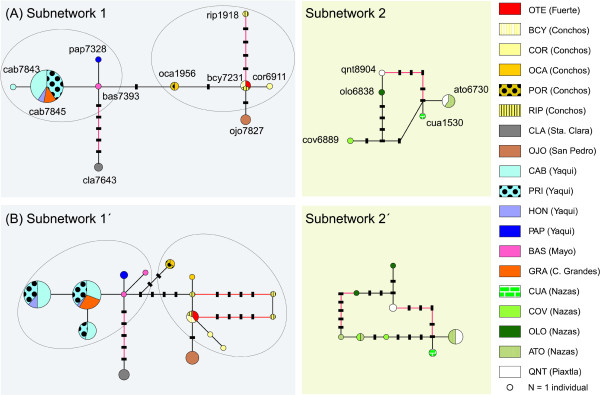
**Statistical parsimony (95%) network for *Campostoma ornatum *S7 nuclear gene haplotypes**. Networks were calculated (a) by treating heterozygous variable sites as unknown data and (b) using reconstructed haplotypes for each allele. Circle size reflects the frequency of each haplotype. Solid lines connecting each pair of haplotypes represent a single mutational event, regardless of their length. Red lines represent indel variation. Small black rectangles represent missing or theoretical haplotypes. The left ellipse groups haplotypes from Yaqui, Casas Grandes and Mayo River basins. The right ellipse gathers sequences from Conchos and Fuerte River basins. Subnetwork 2 (Nazas-Piaxtla) was the most differentiated and is shadowed in green. Abbreviations correspond to names shown in Table 1. River basin names are listed in parentheses.

As a conservative measure, we calculated the 95% SP network for the S7 nuclear gene considering the heterozygous sites as unknown (Figure 4a) (i.e., 15 haplotypes) and with the heterozygotes separated into the different reconstructed haplotypes (Figure 4b, sequences of inferred alleles available from the authors upon request). Differentiation of the Nazas-Aguanaval-Piaxtla River basin (Figure 4) was clearly revealed in both cases (subnetwork 2 and 2'), as individuals from these sites formed one of the two unconnected subnetworks (connection limit = 12). Subnetworks 1 and 1' (Figure 4) were formed by four major groups of haplotypes: Santa Clara, Sonora, Conchos-Fuerte and Yaqui-Casas Grandes-Mayo. Within this subnetwork, Santa Clara was the most differentiated haplogroup, separated by five mutational steps with respect to the BAS haplotype (Mayo basin). These results held regardless of whether gaps were considered as a fifth state.

**Figure 5:**
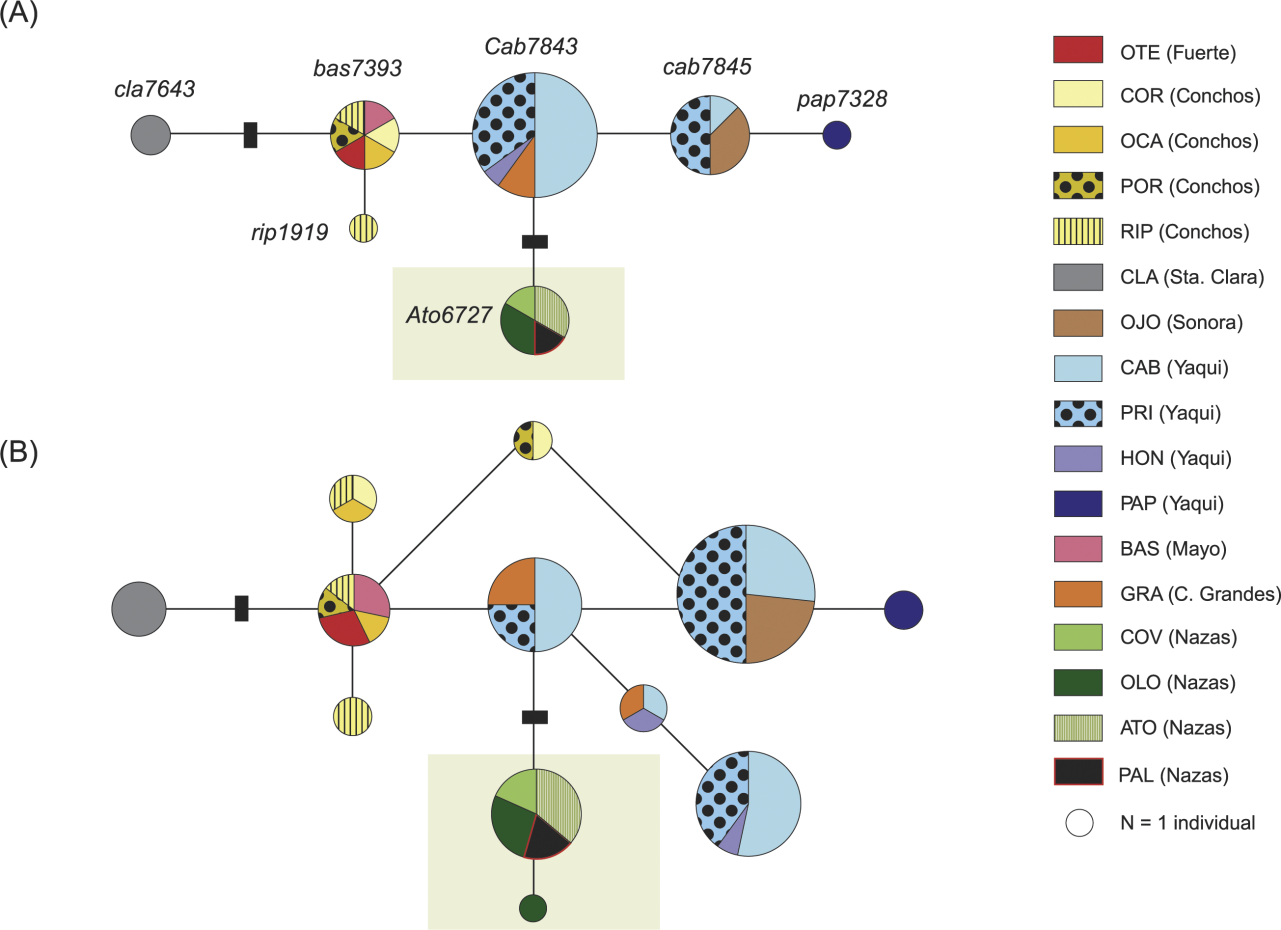
**Statistical parsimony (95%) network for *Campostoma ornatum *RAG1 nuclear gene haplotypes**. Statistical parsimony (95%) networks of 44 individuals of *Campostoma ornatum *sequenced for the nuclear gene RAG1. Networks were calculated (a) by treating heterozygous variable sites as unknown data and (b) using reconstructed haplotypes for each allele. Circle size reflects the frequency of each haplotype. Solid lines connecting each pair of haplotypes represent a single mutational event, regardless of their length. Small black rectangles represent missing or theoretical haplotypes. Haplotypes from Nazas and Aguanaval River basins are shadowed in green. River basin names are listed in parentheses.

The differentiation of the Nazas-Aguanaval River basin was also clearly revealed by the 95% SP network calculated for the RAG1 marker. However, it must be noted that Santa Clara displayed a similar extent of divergence with one intermediate haplotype (Figure 5). Again, sequences of inferred alleles used to calculate Figure 5b are available from the authors upon request.

The estimated age of the most recent common ancestor (TMRCA) of the northern and southern lineages was 3.9 Mya (2.1-5.9). The diversification events within the main northern lineage were dated back to the Lower Pleistocene (Figure 2).

### Morphology

Morphological character values registered in the present study are consistent with the variation pattern obtained by prior literature [24] throughout the range of *C. ornatum*, particularly the counts in lateral line (LLS), predorsal (PrDS) and the circumferential scales (CircS). The most significant result is the distinction between the southern (mitochondrial phylogroup VIII) and northern (I-VII phylogroups) lineages, as revealed by 54-71 LLS (usually 65-66) *vs *62-81 (usually 69-72), 25-32 PrDS (usually 27-29) *vs *29-40 (usually 31-34) and 44-55 CircS (usually 48) vs 50-61 (usually 52-55) (Figure 1 and Additional file 1). Despite the high inter-drainage and inter-population variation recorded, this morphological pattern supports the deep genetic divergence between the Nazas-Aguanaval-Piaxtla basins and the northern drainages (Figures 2, 3, 4, and 5). In contrast, morphological variation within northern group was not congruent with the genetic phylogenies inferred. For instance, specimens from Santa Clara basin (lineage IV) are clearly differentiated from the other northern drainages, regardless of the surveyed genetic marker (Figures 2, 3, 4, and 5). However, their morphological variation appeared overlapped with regard to other lineages within the northern group, e.g., the three surveyed variables fall within the range found for Conchos drainage (Additional file 1).

### Population genetics

Most of the total variation can be explained by the divergence between sampling sites (AMOVA, *Φ*_ST _= 0.92, *p *< 0.001 under the Tamura-Nei model; *Φ*_ST _= 0.58, *p *< 0.001 if only haplotype frequencies are considered) (Table 4). *Φ*_ST_-values were all significant when pairwise comparisons between each phylogroup and between each river basin were tested (Additional files 2 and 3). Additionally, there was a high degree of distinctiveness between locations across river basins as revealed by significant pairwise *Φ*_ST _-values [e.g., *Φ*_ST _= 1 between CON-BCY (Mayo-Conchos), CON-COV (Mayo-Nazas), CON-HUA (Mayo-Yaqui) and BCY-HUA (Conchos-Yaqui)]. Congruently, most of the non-significant values corresponded to intra-basin comparisons [e.g., CON-BAS (Mayo), COR-OCA (Conchos), HON-HUA (Yaqui) and OLO-ATO (Nazas)], but some locations belonging in different drainages also had *Φ*_ST_-values that were not significant [e.g., BCY-URI (Fuerte and Conchos, respectively) and BAS-CON (Mayo and Yaqui, respectively) (Additional file 4)]. Drainage into the Atlantic (the Conchos-Bravo River basin) or to the Pacific Ocean (the remaining river basins) explained much of the genetic variance.

**Table 4:** Analysis of molecular variance (AMOVA) across a range of putative population groupings.

		Variation accounted for (*Φ*)		
		
Grouping criterion	Population grouping	Among groups (*Φ*_CT_*)*	Among populations within groups (*Φ*_SC_*)*	Within populations (*Φ*_ST_*)*
Sampling locality	All in one group	-	0.919***	-
Phylogenetic groups (SP subnetworks)	[I] [II] [III] [IV] [V] [VI] [VII] [VIII]	-	0.891***	-
River basin	[Nazas] [Piaxtla] [Conchos] [Fuerte] [Mayo] [Yaqui] [Sonora] [Casas Grandes] [Santa Clara]	0.704***	0.754***	0.927***
SAMOVA *K *= 4	([Piaxtla+Nazas] [Conchos+Santa Clara+Fuerte] [Yaqui1(Cab, Pap, Pri, Tau, Ter, Tom)+Mayo+Sonora] [Casas Grandes + Yaqui2(Hon, Hua)]	0.692***	0.776***	0.931***
SAMOVA *K *= 13	([Casas Grandes+Yaqui2(Hon, Hua)] [Nazas1(Cov, Pby)] [Mayo+Yaqui1(Tau)] [Nazas2(Ato, Olo)] [Piaxtla] [Conchos1(Por, Sat)] [Conchos2(Cor, Oca, Rip)] [Santa Clara] [Nazas3(Cua)] [Yaqui3(Pap, Ter, Tom)] [Yaqui4(Cab, Pri)] [Conchos3(Bcy)+Fuerte] [Sonora]	0.876***	0.367***	0.921***
Atlantic/Pacific	[Conchos][all other localities]	0.224***	0.913***	0.932***

*** *p *< 0.001. Value in italics corresponds to *Φ*_ST_.

**Figure 6:**
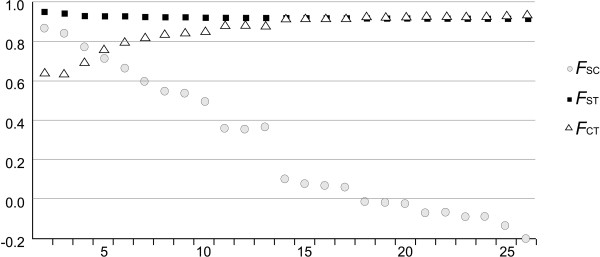
**Spatial analysis of molecular variance (SAMOVA)**. Results of the spatial analysis of molecular variance showing the genetic affinity between Mexican samples of *Campostoma ornatum*. The most likely subdivisions of the whole distribution area were (i) four groups, when the increment of *F*_CT _was the largest (Δ*F*_CT _= 0.06) and (ii) 13 groups, when the decrease of *F*_CT _was maximum (Δ*F*_CT _= - 0.26).

Samples from the Piaxtla, Aguanaval and Nazas River basins clustered together and were clearly separated from the other localities at the lowest *K*-value used for SAMOVA. The population groupings resulting from this analysis at different *K *values closely match the genealogical relationships among haplotypes (Figure 6). We selected *K *= 4 and *K *= 13 as the best groupings, as revealed by the patterns obtained. The clearest rise in *Φ*_CT _values was observed for *K *= 4, which mostly coincided with major lineages obtained in the phylogenetic trees [(Piaxtla+Nazas) (Conchos+Santa Clara+Fuerte) (Yaqui1(Cab, Pap, Pri, Tau, Ter, Tom)+Mayo+Sonora)(Casas Grandes + Yaqui2(Hon, Hua))]. The *Φ*_ST_-values for the *K *= 4 arrangement were all significant, with the highest value resulting from the comparison of Nazas-Aguanaval-Piaxtla with Yaqui2-Casas Grandes (Additional file 5), whereas the largest decrease in *Φ*_SC _was observed when *K *= 13. *F*_CT _values reached the plateau after *K *= 13 (Figure 6). This partition of the data revealed different lineages within the Yaqui, Conchos and Nazas River basins, i.e., [(Casas Grandes+Yaqui2(Hon, Hua))] [Nazas1(Cov, Pby)] [Mayo+Yaqui1(Tau)] [Nazas2(Ato, Olo)] [Piaxtla] [Conchos1(Por, Sat)] [Conchos2(Cor, Oca, Rip)] [Santa Clara] [Nazas3(Cua)] [Yaqui3(Pap, Ter, Tom)] [Yaqui4(Cab, Pri)] [Conchos3(Bcy) +Fuerte] [Sonora]. The *Φ*_ST_-values for the *K *= 13 arrangement were all significant for the comparisons involving Nazas 1 (the Cov and Pby populations) and all other populations, including the Piaxtla River samples (Additional file 6).

Therefore, we analysed the demographic history of mtDNA lineages (i.e., phylogroups in 95% SP unconnected subnetworks), rather than sampling sites or SAMOVA groupings. This way, we could infer which lineages had experienced expansions, instead of trying to infer demographic expansions in the contemporary populations containing haplotypes of mixed ancestry. Neutrality tests applied to each phylogroup revealed no deviations from neutrality due to population expansion. The only lineage with *Fs *and *R2 *statistics close to significance was the one composed of subnetwork V (containing some of the individuals from CAB and PRI, Yaqui River basin) (Table 3). The mismatch distribution analysis for this phylogroup was consistent with the sudden expansion model (SDD = 0.04, *p *= 0.16; raggedness index = 0.32, *p *= 0.12; τ = 0.869, 95% CI = 0-1.818) and showed a unimodal distribution that closely fit the expected distribution (data not shown).

## Discussion

Our genetic and morphological results unambiguously showed two well-differentiated evolutionary groups within *C. ornatum*, supporting prior preliminary investigations [24]. The first lineage is located in the northern river drainages (Yaqui, Mayo, Fuerte, Sonora, Casas Grandes, Santa Clara and Conchos), whereas the other lineage occurs in the southernmost drainages of the species' area of distribution (Nazas, Aguanaval and Piaxtla; central SMOC). The large number of mutation steps between the two lineages exceeded the 95% parsimony limits for the *cytb *and S7 genes, a result indicative of a long history of isolation between these clades. Such an allopatric fragmentation dated back to the Pliocene (3.9 Mya, CI = 2.1-5.9), as revealed by the Bayesian coalescent analyses. The absence of a sharp differentiation between the western (Pacific drainages) and eastern (Atlantic or endorheic basins) lineages, together with the lack of an altitudinal gradient of haplotype diversity (data not shown) lead us to discard a recent (Upper Quaternary) origin for the observed phylogeographic pattern.

In the light of this, and following the proposal of distinguishing between competing models of diversification for North and Mesoamerica [43], we can emphasise tecto-volcanic processes as the crucial factors influencing the evolutionary history of *C. ornatum*, rather than recent habitat fluctuations caused, for instance, by alternating glacial/interglacial stages. The genetic pattern we observed allowed us to infer common river piracy phenomena.

### Southern group

The most plausible biogeographic scenario for the separation of northern and southern groups (mean divergences *P *= 5.2%) is an early Pliocene vicariant event that split a widespread common ancestor. According to our dating, the allopatric fragmentation between the northern and southern lineages took place around 3.9 Mya (CI = 2.2-5.9). This scenario is also supported by the high genetic diversity found in phylogroup V (Naza-Aguanaval-Piaxtla) (Table 3), which is congruent with a high number of female founders followed by long term population stability. Observations from the southern lineage specimens examined in the present study (Nazas and Piaxtla) are in agreement with prior findings [24], as they showed a differentiated morphotype distinguished by higher counts in the lateral, predorsal and circumferential scales, as well as a deeper body. Such a genetic and morphologic differentiation warrants a taxonomic revision likely involving the description of a different taxonomic entity (Figure 2).

The existence of an ancient paleosystem related to the Nazas River has been put forward in prior literature in light of the distribution of its ichthyofauna and herpetofauna [44-46]. Numerous fish species are endemic to the Nazas River [33], which may be indicative of a independent evolutionary history of those species in isolation. Recent molecular surveys also support the existence of an ancient isolation of the Conchos River system and the Nazas, Aguanaval, Mezquital Rivers, a vicariant event that has been dated to *ca*. 5 Mya based on the divergence between the two groups of the genus *Cyprinodon *(Actinopterygii, Ciprinodontiformes) [47]. The ancient connection and disruption of the Nazas palaeosystem with the Conchos River is well supported by the distribution of conspecific species in the *Gila, Cyprinella, Ictiobus *and *Cyprinodon *genera [23,48-50]. Thus, to the best of our knowledge, the presence of *C. ornatum *in the Aguanaval and Piaxtla Rivers is caused by a recent dispersal event from Nazas River, as revealed by the phylogenetic trees, the *cytb *network and *Φ*_ST _results. This hypothesis is also in agreement with the biogeographic scenario postulated for semi-aquatic snakes of the genus *Thamnophis *[46].

### Northern lineage genetic structure and demography

A complex, reticulate biogeographic history in time and space emerged within the northern *C. ornatum *populations, following a clear pattern of taxon pulse episodes [51]. This model assumes that species and their adaptations arise in "centres of diversification" and that distributional ranges of taxa periodically fluctuate around a more stable, continuously occupied centre. This general biotic dispersal may be interrupted by the formation of barriers, producing episodes of vicariant speciation. Breakdown of those barriers produces new episodes of biotic expansion, setting the stage for yet more episodes of vicariance [52]. At this point, it is worth noting the dispersal ability of our model species. We assumed the Mexican stoneroller to be a sedentary species. Its limited active dispersal may putatively be enhanced by environmental factors, such as predation or abiotic change, as has been described for other congeneric species [53,54].

#### Fuerte and Conchos

Although the Fuerte and Conchos River populations form a well-differentiated group (*P *= 2.3% ± 0.4% between Conchos and Fuerte Rivers populations, excluding the BCY and URI populations), pointing to a long evolutionary history in isolation (ca. 1.7 Mya, CI = 0.8-2.9, Figure 2), a recent dispersal event from the Fuerte to Conchos River systems is evident. We base this argument on four lines of evidence: (i) the Urique population (URI, Fuerte basin) shares a haplotype with the Bocoyna population (BCY, Conchos River) (Figure 3), (ii) both locationns have non-significant *Φ*_ST _values, (iii) the position of the URI population in the phylogenetic tree (Figure 2) and (iv) the concordance of the pattern in mitochondrial subnetwork I with the hypothesis of recent (post-glacial) divergence [55]. The close proximity between the headwaters of Conchos and Fuerte River basins makes highly likely the occurrence of a stream capture event between them, as suggested by [24]. Other species co-distributed between these basins, such as *Codoma ornata, Gila pulchra *and *Catostomus plebeius *[23], may have experienced the same dispersal event.

#### Guzman endorheic basin

All markers revealed that the Santa Clara population is the most divergent (mean divergence *p *= 2.9%). This result disagrees with the existence of the hypothesised ancient pluvial Lake Palomas [56,57], which would have covered much of the Guzman Basin systems and would have become fragmented into a series of isolated, endorheic rivers, lakes and springs during the Upper Quaternary (*ca*. 200 000 years ago). This event would have formed the current Guzman basin, which include the Mimbres, Casas Grandes, Santa Maria and Santa Clara River endorheic basins [58]. Our molecular dating showed more ancient episodes of isolation for both the Santa Clara (ca. 2.1 Mya (CI = 1-3.7)) and the Casas Grandes (isolated about 1.5 Mya (CI = 0.4-2.8)) populations (Figure 2). Moreover, the latter was more closely related to samples from the Bavispe River sub-basin (Yaqui River Drainage) than to samples from Santa Clara.

The close relationship between the fish fauna from the Yaqui tributaries and the Casas Grandes system can be attributed to an episode of dispersal from the Casas Grandes to the Yaqui River. This hypothesis is supported by the position of the Yaqui River samples (HON, CAV, HUA and PRI) in the phylogenetic tree (Figure 2) and network (Figure 3), as well as by their low genetic diversity. Such a connection/dispersal event between Yaqui and Casas Grandes is also supported by (i) the occurrence of other co-distributed species (such as *Cyprinella formosa *[58]) and (ii) the finding of very closely related species in each of those river basins (e.g., *Cyprinodon pisteri *and *Cyprinodon albivelis*, formerly considered the same species [59,60], occur at Casas Grandes and Yaqui, respectively [61]).

#### Yaqui basin

Thus far, the Yaqui River supports the most diverse *C. ornatum *populations. Haplotypes found in this northern basin are found in three different mitochondrial subnetworks (including subnetwork VII, as previously explained) (Figure 3) and their supported clades (Figure 2), which was confirmed by the nuclear dataset. Subnetworks V and VI are mainly composed by haplotypes occurring at the Yaqui tributaries and diverged from the other clades around 3 Mya (CI = 1.5-4.9) (Figure 2). This indicates an initial and ancient vicariant event between the Yaqui River and its contiguous basins (Mayo, Sonora and Casas Grandes), followed by episodes of dispersal and subsequent isolation.

Concerning descriptors of genetic diversity, the mitochondrial subnetwork VI had the greatest number of haplotypes, segregating sites, haplotype diversity and average number of differences (Table 3). In light of this, we suggest a dispersal event from the Yaqui to the Mayo River, an event supported by the position of the Mayo populations (BAS and CON) in the phylogenetic tree. Moreover, the La Tauna population (TAU, within Yaqui River) shares a haplotype with the Concheño (CON) and Basaseachic (BAS) populations, both from the Mayo River, and also shows a non-significant pairwise *Φ*_ST _value, which may be indicative of recent gene flow. Faunal exchanges between the Yaqui River and surrounding areas have been previously reported [15,62]. In fact, it has been argued that dispersal between the Yaqui and its contiguous basins was, putatively, a reason for the high fish biodiversity in the Yaqui River [23].

The Yaqui River system hosts three divergent haplogroups (subnetworks V, VI and VII) that occur in sympatry at the Cabullona and Agua Prieta locations (both belonging to Agua Prieta stream, an upper tributary of the Bavispe sub-basin). One of those haplogroups clusters with haplotypes found in the Casas Grandes River forming subnetwork VII. Haplotype cab7845 falls within the second haplogroup (subnetwork VI), closely related with haplotypes form the Tomochic and Papigochic sites (Yaqui River). The third lineage (subnetwork V) is composed by only two haplotypes and shows a mean genetic divergence of *P *= 1.5%, also suggesting a relatively long isolation. Interestingly, the Terapa population (TER), also belonging to the Yaqui River basin, shows a high amount of divergence with respect to other Yaqui populations (*P *= 1.3%). These results are, again, congruent with episodes of isolation in different tributaries of the Yaqui River, followed by a secondary dispersal from some isolated demes to other Yaqui tributaries. We postulate this hypothesis as the most likely explanation for the presence of those three divergent mitochondrial lineages at Cabullona and Agua Prieta.

#### Sonora basin

Haplotypes from the Ojo de Agua (OJO) site, a small spring located at the Sonora River Basin, belong to a separated subnetwork (II) and show large genetic distances (*P *= 2.1% ± 0.3%) with other members of its monophyletic group (Figure 2). This may be indicative of relatively long period of isolation, starting around 1.2 Mya (CI = 0.4-2.2). In addition, the low values of genetic diversity reported for these samples could be caused by a reduction in effective population size, as is expected due to the small size of the sampled area.

### Implications for conservation and taxonomy

In the light of our genetic and morphologic results, we hereby define three ESUs (*sensu *[42]) within *C. ornatum *corresponding to the following groups: (i) the southern lineage, (ii) the northern lineage and (iii) the Yaqui river basin. While acknowledging that the Yaqui belongs to the northern lineage, we define this third sub-hierarchical ESU due to the existence of three deeply divergent lineages at Agua Prieta. Here, the term sub-hierarchical does not infer subordination but rather a frame of reference on a phylogenetic continuum [42]. At present, only two of the sampled localities are currently protected by Mexican legislation (Urique and Huachinera), although the BAS sampling site is close to the Basaseachic Falls National Park. Therefore, the definition of these three ESUs shall promote the conservation of the evolutionary processes shaping the genetic pattern of *C. ornatum *throughout its range.

## Conclusion

We found populations of *C. ornatum *with a complex, reticulate biogeographic history with repeated events of isolation and dispersal, which is consistent with the taxon pulse theory. This complex history could be related to the dynamic tecto-volcanic activity that has occurred in the region since the early Pliocene. Both genetics and morphology revealed a strong differentiation between the southern and northern populations of *C. ornatum*. In light of this, a taxonomic revision is necessary to clarify the taxonomic status of the two well-differentiated groups within *Campostoma ornatum*. The differentiation between the northern and southern taxa was likely due to the isolation of the paleosystem that connected the Nazas with other northern river basins during the Pliocene. In addition, we determined the presence of three very divergent mitochondrial lineages occurring concurrently in the northern Agua Prieta Stream (Yaqui river basin). We postulated that episodes of isolation and the subsequent admixture of lineages from different sources could explain the co-occurrence of these lineages at Yaqui. In addition, three ESUs are proposed that cover the full range of the Mexican stoneroller. The pattern obtained for *Campostoma ornatum *paves the way for a future comparative phylogeographical study aimed at disentangling the evolutionary factors that shape the genetic structure of this species and its populations in the northwest of Mexico.

## Methods

### Specimen collection and laboratory procedures

A total of 285 *Camposoma ornatum *individuals were collected at 29 locations in northwest Mexico (Figure 1). Between 3 and 17 specimens were analysed per location (Table 1). Organisms were caught using a seine net and electrofishing. A small sample of tissue from the caudal fin from each fish was cut and preserved in 96% ethanol and stored in the laboratory at -20°C prior to analysis. Voucher specimens were fixed in 10% formalin and preserved in 70% ethanol and deposited in the *Colección de Peces de la Universidad Michoacana *(CPUM) (Table 1). To confirm the taxonomic identity of *C. ornatum*, morphological examination was performed following [24], i.e., using the three most informative meristic characters: (i) number of lateral line scales, (ii) number of predorsal scales and (iii) number of circumferential scales. We analysed ten specimens corresponding to, at least, one population from each major drainage, except for Aguanaval and Mayo (Additional file 1).

Genomic DNA was extracted from a <0.25 cm^2 ^piece of the pectoral fin using the High Pure™ PCR Template Preparation Kit (Roche Diagnostics GmbH, Mannheim, Germany), following the manufacturer's instructions. We amplified a 1140-bp fragment of the mitochondrial cytochrome b gene (*cytb*) in 285 individuals by Polymerase Chain Reaction (PCR) and the GLUf - THRr primer pair [63].

Amplifications were performed in 30-μL reaction volumes containing 1× PCR buffer (5PRIME), 1 U *Taq *DNA polymerase (5PRIME), 0.2 mM of each dNTP, 0.2 μM of each primer and 50-100 ng of DNA. Similar conditions were employed to amplify the first intron of the S7 ribosomal protein gene in 56 specimens (1000 bp, primers S7RPEX1F and S7RPEX2R [64]) and approximately 1400 bp of the recombinant activation gene 1 (RAG1) in 43 samples (primers RAG1F1 and RAG1R1 [65]).

PCR for *cytb *started with an initial denaturing step at 95°C for 2 minutes, followed by 35 amplification cycles at 94°C for 1 minute, 56°C for 1.5 minutes, 65°C for 1 minute and a final extension step at 65°C for 7 minutes. PCR for S7 started with an initial denaturing step at 95°C for 2 minutes, followed by 30 amplification cycles at 95°C for 1 minute, 59.5°C for 1.5 minutes, 65°C for 2 minutes and a final extension step at 65°C for 7 minutes. PCR for RAG1 started with an initial denaturing step at 94°C for 2 minutes, followed by 40 amplification cycles at 94°C for 45 seconds, 56-58°C for 1 minute, 68°C for 2 minutes and a final extension step at 68°C for 7 minutes.

PCR products were electrophoresed on 2% agarose gels and visualised under UV light after ethidium bromide staining. *Cytb *PCR products were purified and bidirectionally sequenced at the DNA Sequencing Service (Macrogen, Seoul, Korea). S7 and RAG1 PCR products were purified and bidirectionally sequenced on a 3130 × l Genetic Analyser (Applied Biosystems, ABI) at SAI (Sequencing Facility, University of A Coruña, Spain). New sequences were deposited in GenBank (Table 1).

### Alignment and diversity analyses

DNA electropherograms were checked and aligned using CODONCODES 3.5.6 (CodonCode Corporation, Dedham, Massachusetts). Mitochondrial alignments were straightforward. Apart from the 285 individuals sequenced for the present work, phylogenetic analyses included three *Campostoma ornatum cytb *sequences available in public databases for (accession numbers EU082476, DQ324062 and FJ913814). For phylogenetic analyses, RAG1 alignments included sequence EU082549 from Durango (Nazas River basin). Mitochondrial alignments were straightforward and unambiguous. For the analysis of nuclear DNA, the sites where individuals contained heterozygous genotypes for the sampled nuclear loci and any heterozygous base pair positions were coded using standard degeneracy codes [66]. Heterozygous sites in S7 and RAG1 sequences were resolved to two haplotypes per individual using the PHASE feature of DNAsp v.5.1 [67], which uses the algorithms from PHASE 2.1.1 [68]. We employed the recombination model (MR0), setting the output probability for both genotypes and haplotypes to 0.95. Following [69], runs consisted of 500 iterations as burn-in, 500 main iterations and a thinning interval = 1.

Haplotypes and their frequencies, standard indices of genetic variation such as the number of segregating sites (*S*), nucleotide diversity per gene (*π*), haplotype diversity (*h*) and the average number of nucleotide differences (*k*) were calculated using DNAsp 5.1 [67]. Allelic richness (*R*) was computed after rarefaction to three individuals [70] using CONTRIB (available at http://www.pierroton.inra.fr/genetics/labo/Software/) for all populations that included at least three individuals.

### Phylogenetic analyses

As DNA variation may arise through different molecular evolution models, we ran jMODELTEST 0.1.1 [71] to select the one that best matched our three datasets following Akaike's criterion: for the *cytb *gene, we obtained the General Time Reversible model with a gamma distribution shaped with α = 0.175, F81 for S7 and K80+I for RAG1. These models were used to estimate the phylogenetic relationships between all populations of *C. ornatum *based on the mitochondrial *cytb *gene and the S7 and RAG1 nuclear genes. Phylogenetic trees were calculated using the neighbour-joining (NJ), Bayesian inference (BI) and maximum likelihood (ML) methods. Distance analysis was carried out as implemented in PAUP *4.0b10 [72] for 1000 bootstrap replicates. BI was performed in MrBayes [73], and two independent analyses of four MCMC were run with 9,000,000 and 5,000,000 generations for mitochondrial and nuclear loci, respectively, sampling every 100 generations. Once the convergence and stationarity were verified by a suitable effective sample size (ESS) for all parameters in Tracer v.1.4.1 [74], "burn in" consisting in removing the first 10% of generations was performed and posterior probabilities were obtained from a majority-rule consensus. The Maximum Likelihood (ML) phylogeny was inferred using PhyML 3.0 [75] using the subtree pruning and regrafting (SPR) tree searching option. Branch support was assessed after 1000 bootstrap replicates as well as with the Shimodaira-Hasegawa-like (SH) procedure implemented in PhyML. We used *Campostoma oligolepis *(accession number DQ324064) as an outgroup to root the tree. Using sequences from *Campostoma pullum *(EU082477) or *Campostoma pauciradii *(DQ324065) as outgroup did not significantly change any of the results shown in the present study.

The phylogeny of the haplotypes was also inferred using the statistical parsimony (SP) criterion [76]. The 95% SP network was calculated using TCS 1.2 [77]. As several unconnected networks were obtained, we then forced the TCS algorithm to connect all subnetworks with a fixed connection limit of 50 steps. To have a reference to calibrate the extent of divergence between haplogroups, we rooted the resulting network with a homologous sequence from *Campostoma pauciradii *(DQ324065) by forcing a connection limit of 130 steps. Similarly, 95% SP networks were calculated for S7 and RAG1 datasets, both using only homozygous positions (i.e., heterozygous coded as unknown) and the separated haplotypes inferred by PHASE.

### Molecular Clock

We estimated divergence times among sets of mitochondrial sequences and their associated credibility intervals using the Bayesian coalescent approach as implemented in BEAST 1.4.6 [78]. Markov chain Monte Carlo (MCMC) simulations were run with the following specifications: the GTR model of evolution, uncorrelated lognormal distribution, the Yule tree process as a prior and a branch length substitution rate sampled from a prior normal distribution (mean value = 0.010, standard deviation = 0.001). All simulations were run for 50 million generations, sampling chains every 1000 generations. Because of the lack of an appropriate fossil record for the genus *Campostoma*, we applied a rate of molecular evolution of 1.05% per million years, calibrated for the *cytb *in North American Phoxinini [79] and widely applied to cyprinids [14,80]. The results of three independent runs (with an UPGMA starting tree) were loaded and combined in TRACER 1.5 [74] to check for convergence on a stationary distribution, determine burn-in and assess effective sample size (ESS) and frequency plots of the relevant parameters. Ten percent of the trees were discarded as burn-in, and the remaining ones were combined in the BEAST module LogCombiner 1.4.6. Lastly, the BEAST module TreeAnnotator 1.4.6 was used to calculate the timescale.

### Population genetics

We tested for evidence of population subdivision under different criteria using an analysis of molecular variance (AMOVA) as implemented in ARLEQUIN v.3.5.1.2 [81]. We also used ARLEQUIN to test for genetic differentiation between pairs of populations using *F*_ST _analogues (*Φ*_ST_) based on the Tamura-Nei model of sequence evolution. Statistical significance of covariance components (and *Φ*-statistics) from AMOVAs and pairwise *Φ*_ST _values was determined on the basis of the distribution of values obtained from 1000 permutations of the data.

We also assessed population structuring using the simulated annealing procedure as implemented in SAMOVA (Spatial Analysis of Molecular Variance) 1.0 [82]. We used 100 simulated annealing processes for *k *values from 2 to 29.

### Demographic history

The demographic history of the *cytb *phylogroups was first explored using Fu's *Fs *[83] and *R2 *[84] statistics. Their significance was assessed through the 95% confidence interval after 1000 coalescent simulations that were conditional on the number of segregating sites, as implemented in DNAsp.

We characterised the demographic expansions detected for all phylogroups, rejecting the null hypothesis under the *Fs *and *R2 *tests with the mismatch distribution of the pairwise genetic differences [85], as implemented in ARLEQUIN. Goodness-of-fit to a sudden expansion model was tested using parametric bootstrap approaches (1000 replicates). The sum of squared deviations (SSD) between the observed and expected mismatch distributions was used to assess the significance of the test.

## Authors' contributions

ODD conceived the study, collected the samples, performed phylogenetic analyses and drafted the manuscript. MV obtained the DNA sequences, performed the population genetic analyses, participated in the phylogenetic survey and contributed in the writing of the paper. RPR collected the samples, performed morphological and molecular dating analyses and was involved in writing the manuscript. NR carried out the molecular labwork and participated in the sequence alignment. ID conceived the study, participated in its design and coordination as well as was involved in the writing. All authors read and approved the final manuscript.

## Supplementary Material

Additional file 1 **Informative meristic characters for Northern and Southern *Campostoma *groups**. Morphological character values registered throughout the range of *C. ornatum*: counts in lateral line (LLS), predorsal (PrDS) and the circumferential scales (CircS). Click here for file

Additional file 2 **Matrix of population pairwise *Φ*_ST_-values according to phylogenetic grouping**. Matrix of population pairwise *Φ*_ST_-values according to phylogenetic grouping and obtained under the Tamura-Nei model of sequence evolution. All values were significant after correction for multiple testing. Click here for file

Additional file 3 **Matrix of pairwise *Φ*_ST_-values by river basin**. Matrix of pairwise *Φ*_ST_-values by River basin and obtained under the Tamura-Nei model of sequence evolution. All values are significant after correction for multiple testing. Click here for file

Additional file 4 **Matrices of pairwise *Φ*_ST_-values by sampling locality**. Below diagonal: Pairwise *Φ*_ST _values calculated under the Tamura-Nei model. Above diagonal: same calculations using haplotype frequencies. Significance was evaluated after 10000 permutations. Click here for file

Additional file 5 **Matrix of population pairwise *Φ*_ST_-values according to SAMOVA (*K *= 4) groupings**. Matrix of population pairwise *Φ*_ST_-values according to SAMOVA (*K *= 4) groupings and obtained under the Tamura-Nei model of sequence evolution. All values were significant after correction for multiple testing. Click here for file

Additional file 6 **Matrix of population pairwise *Φ*_ST_-values according to SAMOVA (*K *= 13)**. Matrix of population pairwise *Φ*_ST_-values according to SAMOVA (*K *= 13) groupings and obtained under the Tamura-Nei model of sequence evolution. All values significant after correction for multiple testing. Click here for file
