# Supplementary material for: Complex evolutionary history of the Mexican stoneroller Campostoma ornatum Girard, 1856 (Actinopterygii: Cyprinidae)
Source: BMC Evol Biol. 2011 Jun 4;11:153. doi: 10.1186/1471-2148-11-153 (PMC3141424; doi:10.1186/1471-2148-11-153)
Supplement: Additional file 1 — Informative meristic characters for Northern and Southern Campostoma groups. Morphological character values registered throughout the range of C. ornatum: counts in lateral line (LLS), predorsal (PrDS) and the circumferential scales (CircS). [file 1471-2148-11-153-S1.DOC]

**Additional file 1**. Informative meristic characters for Northern and Southern *Campostoma* groups.

|  |  |  |  |  |  |  |  |  |  |  |  |  |  |  |  |  |
| --- | --- | --- | --- | --- | --- | --- | --- | --- | --- | --- | --- | --- | --- | --- | --- | --- |
| **Haplotype** | **Localities** | **Drainages** |  |  |  |  |  | **Meristic scales counts** | | | |  |  |  |  |  |
| **groups** |  |  |  | **LLS** |  |  |  |  | **PrDS** |  |  |  |  | **CircS** |  |  |
|  |  |  | Min/Max | Mean | Mode | N |  | Min/Max | Mean | Mode | N |  | Min/Max | Mean | Mode | N |
|  |  |  |  |  |  |  |  |  |  |  |  |  |  |  |  |  |
| I: Fuerte | Urique River º * | Fuerte | **66-77** | **72.7** | **72(6)** | **24** |  | **30-37** | **33.0** | **32(8)** | **24** |  | **50-57** | **53.6** | **55(7)** | **24** |
|  |  |  |  |  |  |  |  |  |  |  |  |  |  |  |  |  |
| II: Ojo de Agua | Sonora River º | Sonora | **66-74** | **69.8** | **69(6)** | **24** |  | **30-36** | **32.1** | **32(7)** | **18** |  | **52-61** | **55.3** | **55(6)** | **24** |
|  | Ojo de Agua * |  |  |  |  |  |  |  |  |  |  |  |  |  |  |  |
|  |  |  |  |  |  |  |  |  |  |  |  |  |  |  |  |  |
| III: Conchos | Cochos River º | Conchos | **65-81** | **72.0** | **70(21)** | **113** |  | **30-40** | **33.2** | **34(16)** | **79** |  | **50-60** | **56.8** | **54(13)** | **113** |
|  | Florido River º * |  |  |  |  |  |  |  |  |  |  |  |  |  |  |  |
|  | El Porvenir river * |  |  |  |  |  |  |  |  |  |  |  |  |  |  |  |
|  |  |  |  |  |  |  |  |  |  |  |  |  |  |  |  |  |
| IV: Sta. Clara | del Cármen River º | del Cármen | **71-79** | **74.25** | **72(7)** | **24** |  | **30-39** | **32.8** | **33(9)** | **24** |  | **51-59** | **54.6** | **55(5)** | **24** |
|  | Sta. Clara stream * |  |  |  |  |  |  |  |  |  |  |  |  |  |  |  |
|  |  |  |  |  |  |  |  |  |  |  |  |  |  |  |  |  |
| V: Cabullona- | Yaqui River º | Yaqui | **62-80** | **70.5** | **72(10)** | **63** |  | **30-36** | **32.0** | **31(16)** | **54** |  | **51-59** | **53,8** | **52(15)** | **62** |
| San Bernardino | San Bernardino River * |  |  |  |  |  |  |  |  |  |  |  |  |  |  |  |
|  |  |  |  |  |  |  |  |  |  |  |  |  |  |  |  |  |
| VI: Yaqui-Mayo | Basaseachic River * | Mayo | **70-74** | **71.2** | **70(3)** | **5** |  | **29-31** | **30.6** | **31(4)** | **5** |  | **53-55** | **53.4** | **55(4)** | **5** |
|  |  |  |  |  |  |  |  |  |  |  |  |  |  |  |  |  |
| VII: Yaqui- | Casas grandes River º* | Casas grandes | **62-74** | **74.2** | **70(6)** | **36** |  | **30-34** | **31.6** | **31(15)** | **36** |  | **50-57** | **55.2** | **54(9)** | **36** |
| Casas Grandes |  |  |  |  |  |  |  |  |  |  |  |  |  |  |  |  |
|  |  |  |  |  |  |  |  |  |  |  |  |  |  |  |  |  |
| VIII:Nazas- | Nazas River º | Nazas | **54-71** | **63.5** | **65(11)** | **71** |  | **25-32** | **28.8** | **29(18)** | **71** |  | **44-55** | **45.75** | **48(17)** | **71** |
| Aguanval-Piaxtla | Peñon Blanco * |  |  |  |  |  |  |  |  |  |  |  |  |  |  |  |
|  |  |  |  |  |  |  |  |  |  |  |  |  |  |  |  |  |
|  | Miravalle º | Piaxtla | **56-69** | **63.1** | **66(5)** | **24** |  | **25-31** | **28.1** | **27(7)** | **18** |  | **45-50** | **47.8** | **48(8)** | **24** |
|  | Stream at Rancho |  |  |  |  |  |  |  |  |  |  |  |  |  |  |  |
|  | La Quinta * |  |  |  |  |  |  |  |  |  |  |  |  |  |  |  |
|  |  |  |  |  |  |  |  |  |  |  |  |  |  |  |  |  |
| º = obtained from Burr (1976). | |  | LLS= lateral line scales; PrDS= predorsal scales; CircS= circumferential scales. | | | | | | | | | | | | |  |
| * = obtained from populations with specimens available. | | | Numbers in parenthesis correspond to number of specimens. | | | | | | | | | | |  |  |  |
