# Supplementary material for: Complex evolutionary history of the Mexican stoneroller Campostoma ornatum Girard, 1856 (Actinopterygii: Cyprinidae)
Source: BMC Evol Biol. 2011 Jun 4;11:153. doi: 10.1186/1471-2148-11-153 (PMC3141424; doi:10.1186/1471-2148-11-153)
Supplement: Additional file 2 — Matrix of population pairwise ΦST-values according to phylogenetic grouping. Matrix of population pairwise ΦST-values according to phylogenetic grouping and obtained under the Tamura-Nei model of sequence evolution. All values were significant after correction for multiple testing. [file 1471-2148-11-153-S2.DOC]

**Aditional file 2**. Matrix of population pairwise *Φ*_ST_-values according to phylogenetic grouping (95% SP unconnected subnetworks) and obtained under the Tamura-Nei model of sequence evolution. All values were significant after correction for multiple testing (1023 permutations; adjusted alpha-value = 0.0018). Numbers of individuals analysed within phylogroup are displayed in parentheses. The three largest values are highlighted in bold, the three lowest values are marked in italics.

|  | I (44) | II | III | IV | V | VI | VII |
| --- | --- | --- | --- | --- | --- | --- | --- |
| II (10) | **0.971** |  |  |  |  |  |  |
| III (30) | 0.945 | 0.957 |  |  |  |  |  |
| IV (10) | 0.952 | 0.961 | 0.941 |  |  |  |  |
| V (10) | **0.970** | **0.966** | 0.959 | 0.966 |  |  |  |
| VI (76) | 0.802 | 0.647 | 0.785 | *0.755* | *0.581* |  |  |
| VII (48) | 0.941 | 0.944 | 0.940 | 0.935 | 0.942 | *0.796* |  |
| VIII (57) | 0.932 | 0.917 | 0.931 | 0.920 | 0.919 | 0.869 | 0.935 |
