# Supplementary material for: Complex evolutionary history of the Mexican stoneroller Campostoma ornatum Girard, 1856 (Actinopterygii: Cyprinidae)
Source: BMC Evol Biol. 2011 Jun 4;11:153. doi: 10.1186/1471-2148-11-153 (PMC3141424; doi:10.1186/1471-2148-11-153)
Supplement: Additional file 3 — Matrix of pairwise ΦST-values by river basin. Matrix of pairwise ΦST-values by River basin and obtained under the Tamura-Nei model of sequence evolution. All values are significant after correction for multiple testing. [file 1471-2148-11-153-S3.DOC]

**Additional file 3.** Matrix of pairwise *Φ*_ST_-values by River basin and obtained under the Tamura-Nei model of sequence evolution. All values are significant after correction for multiple testing (1023 permutations; adjusted alpha-value = 0.0014). Numbers of individuals analysed within each River basin are displayed in parentheses. The three largest values are highlighted in bold, the three lowest values are marked in italics.

|  | Nazas (47) | Piaxtla | Conchos | Fuerte | Mayo | Yaqui | Sonora | Casas Grandes |
| --- | --- | --- | --- | --- | --- | --- | --- | --- |
| Piaxtla (10) | *0.374* |  |  |  |  |  |  |  |
| Conchos (40) | 0.867 | 0.865 |  |  |  |  |  |  |
| Fuerte (34) | 0.929 | 0.980 | 0.675 |  |  |  |  |  |
| Mayo (21) | 0.933 | **0.999** | 0.779 | 0.969 |  |  |  |  |
| Yaqui (93) | 0.761 | 0.737 | 0.514 | 0.586 | *0.306* |  |  |  |
| Sonora (10) | 0.917 | **0.989** | 0.785 | 0.967 | 0.980 | *0.447* |  |  |
| Casas Grandes (20) | 0.928 | 0.983 | 0.774 | 0.949 | 0.972 | *0.447* | 0.958 |  |
| Santa Clara (10) | 0.921 | **0.984** | 0.724 | 0.944 | 0.978 | 0.551 | 0.961 | 0.947 |
