# Supplementary material for: Complex evolutionary history of the Mexican stoneroller Campostoma ornatum Girard, 1856 (Actinopterygii: Cyprinidae)
Source: BMC Evol Biol. 2011 Jun 4;11:153. doi: 10.1186/1471-2148-11-153 (PMC3141424; doi:10.1186/1471-2148-11-153)
Supplement: Additional file 4 — Matrices of pairwise ΦST-values by sampling locality. Below diagonal: Pairwise ΦST values calculated under the Tamura-Nei model. Above diagonal: same calculations using haplotype frequencies. Significance was evaluated after 10000 permutations. [file 1471-2148-11-153-S4.DOC]

**Additional file 4.** Below diagonal: Pairwise *Φ*_ST_ values calculated under the Tamura-Nei model (assuming action of drift and mutation). Above diagonal: same calculations using haplotype frequencies (assuming genetic drift to be the only force acting in diversifying populations). Significance was evaluated after 10,000 permutations. Values underlined remained not significant after Bonferroni correction (adjusted nominal value = 0.00006). Values significant after correction for multiple testing are displayed in bold. Negative values are due to algorithm application and should be regarded as zero.

|  | ATO | BAS | BCY | CAB | CLA | CON | COR | COV | CUA | GRA | HON | HUA | OCA | OJO | OLO | OTE | PAP | PBY | POR | PRI | QNT | RIM | RIP | SAT | TAU | TER | TOM | URI | ZAR |
| --- | --- | --- | --- | --- | --- | --- | --- | --- | --- | --- | --- | --- | --- | --- | --- | --- | --- | --- | --- | --- | --- | --- | --- | --- | --- | --- | --- | --- | --- |
| ATO |  | 0.63 | 0.73 | 0.36 | 0.33 | 0.74 | 0.65 | 0.66 | 0.56 | 0.41 | 0.63 | 0.73 | 0.43 | 0.39 | 0.02 | 0.66 | 0.66 | 0.65 | 0.54 | 0.39 | 0.70 | 0.39 | 0.60 | 0.43 | 0.41 | 0.40 | 0.35 | 0.76 | 0.37 |
| BAS | 0.99 |  | 0.90 | 0.50 | 0.50 | 0.01 | 0.81 | 0.87 | 0.72 | 0.58 | 0.80 | 0.90 | 0.69 | 0.56 | 0.45 | 0.82 | 0.82 | 0.81 | 0.71 | 0.52 | 0.90 | 0.56 | 0.84 | 0.69 | 0.34 | 0.56 | 0.53 | 0.91 | 0.53 |
| BCY | 0.99 | 1 |  | 0.58 | 0.60 | 1 | 0.91 | 1 | 0.82 | 0.68 | 0.90 | 1 | 0.86 | 0.66 | 0.55 | 0.91 | 0.91 | 0.91 | 0.81 | 0.60 | 1 | 0.66 | 1 | 0.86 | 0.68 | 0.65 | 0.64 | 0 | 0.63 |
| CAB | 0.85 | 0.36 | 0.68 |  | 0.24 | 0.59 | 0.52 | 0.51 | 0.43 | 0.31 | 0.50 | 0.58 | 0.29 | 0.29 | 0.20 | 0.53 | 0.53 | 0.52 | 0.42 | 0.08 | 0.58 | 0.29 | 0.45 | 0.29 | 0.31 | 0.30 | 0.24 | 0.60 | 0.27 |
| CLA | 0.98 | 0.97 | 0.97 | 0.69 |  | 0.61 | 0.52 | 0.50 | 0.42 | 0.28 | 0.50 | 0.60 | 0.24 | 0.26 | 0.16 | 0.54 | 0.54 | 0.52 | 0.41 | 0.27 | 0.60 | 0.26 | 0.43 | 0.24 | 0.28 | 0.27 | 0.21 | 0.63 | 0.23 |
| CON | 0.99 | 0.01 | 1 | 0.37 | 0.98 |  | 0.91 | 1 | 0.83 | 0.69 | 0.91 | 1 | 0.87 | 0.67 | 0.56 | 0.91 | 0.91 | 0.91 | 0.82 | 0.61 | 1 | 0.67 | 1 | 0.87 | 0.48 | 0.66 | 0.66 | 1 | 0.65 |
| COR | 0.99 | 0.99 | 0.99 | 0.68 | 0.97 | 0.99 |  | 0.87 | 0.74 | 0.60 | 0.81 | 0.91 | 0.11 | 0.57 | 0.47 | 0.83 | 0.83 | 0.82 | 0.69 | 0.54 | 0.91 | 0.57 | -0.19 | 0.71 | 0.60 | 0.57 | 0.55 | 0.91 | 0.55 |
| COV | 0.85 | 1 | 1 | 0.81 | 0.99 | 1 | 1 |  | 0.77 | 0.59 | 0.87 | 1 | 0.76 | 0.57 | 0.45 | 0.88 | 0.88 | -0.09 | 0.75 | 0.53 | 1 | 0.57 | 1 | 0.76 | 0.59 | 0.57 | 0.54 | 1 | 0.54 |
| CUA | 0.87 | 1 | 1 | 0.85 | 0.99 | 1 | 0.99 | 0.89 |  | 0.50 | 0.72 | 0.82 | 0.56 | 0.48 | 0.38 | 0.75 | 0.75 | 0.74 | 0.63 | 0.46 | 0.82 | 0.48 | 0.73 | 0.56 | 0.50 | 0.48 | 0.44 | 0.84 | 0.46 |
| GRA | 0.98 | 0.98 | 0.98 | 0.57 | 0.96 | 0.98 | 0.98 | 0.99 | 0.99 |  | 0.58 | 0.68 | 0.35 | 0.33 | 0.24 | 0.61 | 0.61 | 0.60 | 0.49 | 0.34 | 0.68 | 0.33 | 0.53 | 0.35 | 0.36 | 0.34 | 0.29 | 0.70 | 0.28 |
| HON | 0.99 | 1 | 1 | 0.57 | 0.97 | 1 | 0.99 | 1 | 1 | 0.76 |  | 0 | 0.69 | 0.56 | 0.45 | 0.82 | 0.82 | 0.81 | 0.71 | 0.52 | 0.90 | 0.56 | 0.84 | 0.69 | 0.58 | 0.56 | 0.53 | 0.91 | 0.53 |
| HUA | 0.99 | 1 | 1 | 0.57 | 0.97 | 1 | 0.99 | 1 | 1 | 0.79 | 0 |  | 0.86 | 0.66 | 0.55 | 0.91 | 0.91 | 0.91 | 0.81 | 0.60 | 1 | 0.66 | 1 | 0.86 | 0.68 | 0.65 | 0.64 | 1 | 0.63 |
| OCA | 0.97 | 0.98 | 0.98 | 0.55 | 0.94 | 0.98 | 0.16 | 0.99 | 0.99 | 0.96 | 0.98 | 0.99 |  | 0.32 | 0.20 | 0.73 | 0.73 | 0.71 | 0.51 | 0.33 | 0.86 | 0.32 | 0 | 0.33 | 0.35 | 0.33 | 0.26 | 0.88 | 0.29 |
| OJO | 0.98 | 0.97 | 0.99 | 0.55 | 0.97 | 0.98 | 0.98 | 0.99 | 0.99 | 0.97 | 0.98 | 0.99 | 0.97 |  | 0.22 | 0.59 | 0.59 | 0.57 | 0.47 | 0.32 | 0.66 | 0.31 | 0.50 | 0.32 | 0.33 | 0.32 | 0.27 | 0.68 | 0.29 |
| OLO | -0.03 | 0.97 | 0.97 | 0.84 | 0.97 | 0.97 | 0.97 | 0.65 | 0.72 | 0.97 | 0.97 | 0.97 | 0.95 | 0.97 |  | 0.49 | 0.49 | 0.47 | 0.37 | 0.23 | 0.50 | 0.22 | 0.38 | 0.20 | 0.24 | 0.23 | 0.17 | 0.58 | 0.20 |
| OTE | 0.99 | 1 | 0.92 | 0.71 | 0.97 | 1 | 0.99 | 1 | 1 | 0.98 | 1 | 1 | 0.98 | 0.99 | 0.97 |  | 0.83 | 0.83 | 0.74 | 0.55 | 0.91 | 0.59 | 0.86 | 0.73 | 0.61 | 0.59 | 0.57 | 0.92 | 0.57 |
| PAP | 0.99 | 0.98 | 1 | 0.32 | 0.98 | 0.99 | 0.99 | 1 | 1 | 0.98 | 0.99 | 1 | 0.98 | 0.97 | 0.97 | 1 |  | 0.83 | 0.74 | 0.55 | 0.91 | 0.59 | 0.86 | 0.73 | 0.61 | 0.59 | 0.16 | 0.92 | 0.57 |
| PBY | 0.88 | 1 | 1 | 0.86 | 0.99 | 1 | 1 | -0.09 | 0.89 | 0.99 | 1 | 1 | 0.99 | 0.99 | 0.73 | 1 | 1 |  | 0.72 | 0.54 | 0.90 | 0.57 | 0.85 | 0.71 | 0.60 | 0.57 | 0.55 | 0.91 | 0.55 |
| POR | 0.98 | 0.99 | 0.99 | 0.66 | 0.96 | 0.99 | 0.71 | 0.99 | 0.99 | 0.98 | 0.99 | 0.99 | 0.55 | 0.98 | 0.97 | 0.99 | 0.99 | 1 |  | 0.45 | 0.81 | 0.47 | 0.68 | 0.54 | 0.49 | 0.47 | 0.43 | 0.83 | 0.44 |
| PRI | 0.85 | 0.36 | 0.69 | -0.06 | **0.69** | 0.37 | 0.69 | 0.82 | 0.85 | 0.60 | 0.60 | 0.60 | 0.56 | 0.55 | 0.84 | 0.72 | 0.27 | 0.86 | 0.66 |  | 0.60 | 0.32 | 0.48 | 0.33 | 0.34 | 0.33 | 0.28 | 0.62 | 0.30 |
| QNT | 0.27 | 1 | 1 | 0.85 | 0.99 | 1 | 1 | 1 | 0.98 | 0.99 | 1 | 1 | 0.99 | 0.99 | 0.16 | 1 | 1 | 0.99 | 1 | 0.86 |  | 0.66 | 1 | 0.86 | 0.68 | 0.65 | 0.64 | 1 | 0.63 |
| RIM | 0.98 | 0.98 | 0.72 | 0.67 | 0.96 | 0.99 | 0.97 | 0.99 | 0.99 | 0.97 | 0.98 | 0.99 | 0.95 | 0.98 | 0.96 | 0.81 | 0.99 | 0.99 | 0.97 | 0.67 | 0.99 |  | 0.50 | 0.32 | 0.33 | 0.32 | 0.27 | 0.68 | 0.29 |
| RIP | 0.98 | 1 | 1 | 0.57 | 0.96 | 1 | -0.19 | 1 | 1 | 0.98 | 1 | 1 | 0 | 0.98 | 0.96 | 1 | 0.99 | 1 | 0.78 | 0.59 | 1 | 0.97 |  | 0.67 | 0.53 | 0.51 | 0.46 | 1 | 0.47 |
| SAT | 0.98 | 0.99 | 0.99 | 0.55 | 0.95 | 1 | 0.51 | 1 | 1 | 0.97 | 0.99 | 1 | 0.05 | 0.98 | 0.95 | 0.99 | 0.99 | 1 | 0.56 | 0.56 | 1 | 0.97 | 0.75 |  | 0.35 | 0.33 | 0.26 | 0.88 | 0.29 |
| TAU | 0.98 | 0.37 | 0.99 | 0.37 | 0.96 | 0.43 | 0.98 | 0.99 | 0.99 | 0.97 | 0.99 | 0.99 | 0.96 | 0.96 | 0.96 | 0.99 | 0.96 | 0.99 | 0.98 | 0.37 | 1 | 0.98 | 0.98 | 0.98 |  | 0.34 | 0.29 | 0.70 | 0.31 |
| TER | 0.98 | 0.95 | 0.98 | 0.52 | 0.96 | 0.95 | 0.97 | 0.99 | 0.99 | 0.97 | 0.98 | 0.98 | 0.95 | 0.95 | 0.96 | 0.98 | 0.93 | 0.99 | 0.97 | 0.51 | 0.99 | 0.97 | 0.97 | 0.96 | 0.93 |  | 0.28 | 0.68 | 0.30 |
| TOM | 0.98 | 0.96 | 0.99 | 0.27 | 0.97 | 0.97 | 0.98 | 0.99 | 0.99 | 0.97 | 0.99 | 0.99 | 0.96 | 0.95 | 0.97 | 0.99 | 0.02 | 1 | 0.98 | 0.22 | 1 | 0.98 | 0.98 | 0.98 | 0.94 | 0.91 |  | 0.67 | 0.24 |
| URI | 0.99 | 1 | 0 | 0.70 | 0.97 | 1 | 0.99 | 1 | 1 | 0.98 | 1 | 1 | 0.98 | 0.99 | 0.97 | 0.92 | 1 | 1 | 0.99 | 0.71 | 1 | 0.74 | 1 | 1 | 0.99 | 0.98 | 0.99 |  | 0.66 |
| ZAR | 0.98 | 0.97 | 0.97 | 0.56 | 0.95 | 0.98 | 0.97 | 0.99 | 0.99 | 0.08 | 0.69 | 0.71 | 0.95 | 0.97 | 0.97 | 0.98 | 0.98 | 0.99 | 0.97 | 0.59 | 0.99 | 0.96 | 0.97 | 0.96 | 0.96 | 0.96 | 0.97 | 0.98 |  |
