# Supplementary material for: Complex evolutionary history of the Mexican stoneroller Campostoma ornatum Girard, 1856 (Actinopterygii: Cyprinidae)
Source: BMC Evol Biol. 2011 Jun 4;11:153. doi: 10.1186/1471-2148-11-153 (PMC3141424; doi:10.1186/1471-2148-11-153)
Supplement: Additional file 5 — Matrix of population pairwise ΦST-values according to SAMOVA (K = 4) groupings. Matrix of population pairwise ΦST-values according to SAMOVA (K = 4) groupings and obtained under the Tamura-Nei model of sequence evolution. All values were significant after correction for multiple testing. [file 1471-2148-11-153-S5.DOC]

**Additional file 5**. Matrix of population pairwise *Φ*_ST_-values according to SAMOVA (*K* = 4) groupings and obtained under the Tamura-Nei model of sequence evolution. All values were significant after correction for multiple testing (1023 permutations; adjusted alpha-value = 0.0083). Numbers of individuals analysed within each grouping are displayed in parentheses. The largest value is highlighted in bold, the lowest one is marked in italics. Yaqui1 includes de following localities: CAB, PAP, PRI, TAU, TER, and TOM. Yaqui2 includes HON and HUA.

|  | Nazas-Aguanaval- Piaxtla (57) | Conchos-Santa Clara-Fuerte | Yaqui1-Mayo-Sonora |
| --- | --- | --- | --- |
| Conchos-Santa Clara-Fuerte (84) | 0.799 |  |  |
| Yaqui1-Mayo-Sonora (104) | 0.801 | *0.519* |  |
| Yaqui2-Casas Grandes (40) | **0.933** | 0.646 | 0.636 |
