# Supplementary material for: Complex evolutionary history of the Mexican stoneroller Campostoma ornatum Girard, 1856 (Actinopterygii: Cyprinidae)
Source: BMC Evol Biol. 2011 Jun 4;11:153. doi: 10.1186/1471-2148-11-153 (PMC3141424; doi:10.1186/1471-2148-11-153)
Supplement: Additional file 6 — Matrix of population pairwise ΦST-values according to SAMOVA (K = 13). Matrix of population pairwise ΦST-values according to SAMOVA (K = 13) groupings and obtained under the Tamura-Nei model of sequence evolution. All values significant after correction for multiple testing. [file 1471-2148-11-153-S6.DOC]

**Additional file 6.** Matrix of population pairwise *Φ*_ST_-values according to SAMOVA (*K* = 13) groupings and obtained under the Tamura-Nei model of sequence evolution. All values significant after correction for multiple testing (1023 permutations; adjusted alpha-value = 0.0083). Numbers of individuals analysed within each grouping are displayed in parentheses. The largest value is highlighted in bold, the lowest one is marked in italics. CG: Casas Grandes (GRA), Mayo: BAS, CO; Yaqui1 includes: TAU; Yaqui2 includes HON and HUA; Yaqui3:PAP, TER, TOM; Yaqui4: CAB, PRI; Nazas1: COV, PBY; Nazas2: ATO, OLO; Nazas3: CUA; Piaxtla: QNT; Conchos1: POR, SAT; Conchos2: COR, OCA, RIP; Conchos3: BCY; Fuerte: URI, RIM, OTE; Sonora: OJO.

|  | CasasGrandes_  Yaqui2 (40) | Nazas1 | Yaqui1_  Mayo | Nazas2 | Piaxtla | Conchos1 | Conchos2 | Santa Clara | Nazas3 | Yaqui3 | Yaqui4 | Conchos3_  Fuerte |
| --- | --- | --- | --- | --- | --- | --- | --- | --- | --- | --- | --- | --- |
| Nazas1 (16) | 0.975 |  |  |  |  |  |  |  |  |  |  |  |
| Yaqui1_Mayo (31) | 0.955 | **0.994** |  |  |  |  |  |  |  |  |  |  |
| Nazas2 (21) | 0.960 | 0.783 | 0.971 |  |  |  |  |  |  |  |  |  |
| Piaxtla (10) | 0.974 | **0.991** | 0.993 | *0.152* |  |  |  |  |  |  |  |  |
| Conchos1 (13) | 0.946 | **0.991** | 0.978 | 0.958 | 0.989 |  |  |  |  |  |  |  |
| Conchos2 (17) | 0.949 | **0.991** | 0.978 | 0.962 | 0.989 | 0.566 |  |  |  |  |  |  |
| Santa_Clara (10) | 0.938 | 0.987 | 0.973 | 0.952 | 0.984 | 0.951 | 0.956 |  |  |  |  |  |
| Nazas3 (10) | 0.972 | 0.906 | **0.992** | 0.747 | 0.981 | 0.987 | 0.987 | 0.981 |  |  |  |  |
| Yaqui3 (31) | 0.867 | 0.917 | 0.728 | 0.904 | 0.907 | 0.837 | 0.852 | 0.822 | 0.904 |  |  |  |
| Yaqui4 (32) | 0.632 | 0.804 | *0.477* | 0.804 | 0.786 | 0.629 | 0.663 | 0.628 | 0.780 | *0.276* |  |  |
| Conchos3_Fuerte (44) | 0.945 | 0.984 | 0.970 | 0.965 | 0.983 | 0.954 | 0.957 | 0.952 | 0.982 | 0.887 | 0.734 |  |
| Sonora (10) | 0.947 | **0.991** | 0.971 | 0.954 | 0.989 | 0.969 | 0.972 | 0.961 | 0.986 | 0.733 | 0.515 | 0.971 |
